# Pangenome-Wide Association Study and Transcriptome Analysis Reveal a Novel QTL and Candidate Genes Controlling both Panicle and Leaf Blast Resistance in Rice

**DOI:** 10.1186/s12284-024-00707-x

**Published:** 2024-04-12

**Authors:** Jian Wang, Haifei Hu, Xianya Jiang, Shaohong Zhang, Wu Yang, Jingfang Dong, Tifeng Yang, Yamei Ma, Lian Zhou, Jiansong Chen, Shuai Nie, Chuanguang Liu, Yuese Ning, Xiaoyuan Zhu, Bin Liu, Jianyuan Yang, Junliang Zhao

**Affiliations:** 1 Rice Research Institute, Guangdong Academy of Agricultural Sciences & Key Laboratory of Genetics and Breeding of High Quality Rice in Southern China (Co-construction by Ministry and Province), Ministry of Agriculture and Rural Affairs & Guangdong Key Laboratory of New Technology in Rice Breeding & Guangdong Rice Engineering Laboratory, Guangzhou, 510640 China; 2 Plant Protection Research Institute, Guangdong Academy of Agricultural Sciences & Guangdong Provincial Key Laboratory of High Technology for Plant Protection, Guangzhou, 510640 China; 3 Yangjiang Institute of Agricultural Sciences, Yangjiang, 529500 China; 4 State Key Laboratory for Biology of Plant Diseases and Insect Pests, Institute of Plant Protection, Chinese Academy of Agricultural Sciences, Beijing, 100193 China

**Keywords:** Rice, Blast resistance, Pangenome-wide association study, Transcriptomic analysis, Candidate genes

## Abstract

Cultivating rice varieties with robust blast resistance is the most effective and economical way to manage the rice blast disease. However, rice blast disease comprises leaf and panicle blast, which are different in terms of resistance mechanisms. While many blast resistant rice cultivars were bred using genes conferring resistance to only leaf or panicle blast, mining durable and effective quantitative trait loci (QTLs) for both panicle and leaf blast resistance is of paramount importance. In this study, we conducted a pangenome-wide association study (panGWAS) on 9 blast resistance related phenotypes using 414 international diverse rice accessions from an international rice panel. This approach led to the identification of 74 QTLs associated with rice blast resistance. One notable locus, *qPBR1*, validated in a F_4:5_ population and fine-mapped in a Heterogeneous Inbred Family (HIF), exhibited broad-spectrum, major and durable blast resistance throughout the growth period. Furthermore, we performed transcriptomic analysis of 3 resistant and 3 sensitive accessions at different time points after infection, revealing 3,311 differentially expressed genes (DEGs) potentially involved in blast resistance. Integration of the above results identified 6 candidate genes within the *qPBR1* locus, with no significant negative effect on yield. The results of this study provide valuable germplasm resources, QTLs, blast response genes and candidate functional genes for developing rice varieties with enduring and broad-spectrum blast resistance. The *qPBR1*, in particular, holds significant potential for breeding new rice varieties with comprehensive and durable resistance throughout their growth period.

**Supplementary Information:**

The online version contains supplementary material available at 10.1186/s12284-024-00707-x.

## Background

Rice blast disease, caused by the ascomycete fungal pathogen *Magnaporthe oryzae*, is one of the most devastating diseases resulting in significant global rice production losses. Annually, rice blast disease accounts for an estimated $66 billion in rice losses, sufficient to feed 60 million people (Pennisi 2010). In recent years, rice blast has become increasingly severe due to climate change resulting in increases in temperature and precipitation, underscoring the urgent need to cultivate new rice varieties with comprehensive and durable rice blast resistance to ensure global food security.

In response to microbial pathogens carrying effectors, rice has evolved a multifaceted and sophisticated innate immune system. This defense mechanism initiates with pattern recognition of receptors mediating to pathogen elicitors during pathogen attachment and the early phase of host-pathogen interactions. Subsequently, plant resistance (*R*) genes mediate the effector-triggered immunity, playing a pivotal role in establishing resistance. Major *R* genes encoding cytoplasmic proteins with nucleotide binding site-leucine-rich repeat (NLR) domains (Dangl et al. 2013), represent a unique mode in evolution, and are considered as the core component of the plant immune system (Clark et al. 2007). At present, over 100 rice blast resistance (*R*) genes have been identified, with more than 30 genes being molecularly cloned (Singh et al. 2018; Zhao et al. 2018a). However, blast mainly appears in two forms, the leaf blast and the panicle (or neck) blast, both causing major rice yield losses. Most of the studies have predominantly focused on leaf blast resistance (Qu et al. 2006; Lin et al. 2007), leaving the genetic basis and molecular mechanisms of panicle blast resistance largely unknown. Therefore, it is of utmost importance to have a thorough comprehension of the genetic factors underlying the differences between leaf and panicle blast resistance.

Due to the race-specific effects of *R* genes, most of the studies have focused on identifying new *R* genes based on a few races of the rice blast fungus (Bryan et al. 2000; Zhou et al. 2019). While rice resistance can be improved through the use of individual *R* gene or by pyramiding multiple *R* genes, the continuous genetic evolution of *M. oryzae* presents a significant obstacle for molecular breeding efforts targeted toward enhancing blast resistance in this staple crop. The pathogen’s genetic variability and loss of avirulence (AVR) genes can circumvent plant defenses, often leading to the breakdown of resistance in rice varieties within a few years (Dean et al. 2005).

Therefore, identifying QTLs/genes with resistance to both panicle and leaf blast, as well as to broad-spectrum of blast isolates is one of the most effective strategies for breeding new rice cultivars with sustained blast resistance. Traditionally, the identification of resistant genes relied on map-based cloning utilizing extensive segregating populations, such as F_2_ population, recombinant inbred lines (RILs) and doubled haploid populations (Takagi et al. 2013). These methods are constrained by the genetic variation present among the mapping parents, thereby restricting the analysis to only two allelic variations. Moreover, they are laborious and time-consuming. With the reduction in high-throughput sequencing costs, genome-wide association studies (GWAS) have emerged as a potent tool for exploring multiple genetic variations underlying complex phenotypes (Yano et al. 2016; Zhao et al. 2018b). A recent study we conducted involved the creation of a rice pangenome and its use as a reference for GWAS. Our results showed a significant improvement in the ability to detect causal variants by GWAS using our constructed pangenpome (Wang et al. 2023). Additionally, integrating GWAS with transcriptomic profiling further improve the ability for pinpointing genes associated with specific traits, as evidenced by the identification of genes such as *OsSPL13* (Si et al. 2016), *GL7* (Wang et al. 2015), and *pid4* (Chen et al. 2018).

To address the challenges posed by the limited understanding of panicle blast and the absence of broad-spectrum resistance genes, we conducted a pangenome GWAS (Pan-GWAS) using a diverse rice accession panel consisting of 414 international rice accessions and our previous pangenome (Wang et al. 2023). By assessing the resistance phenotype of both panicle and leaf blast in the nursery, as well as inoculating 7 representative isolates in green house, novel QTLs conferring panicle or leaf blast resistance in rice were identified. One of the most significant QTL (*qPBR1*) that confers resistance to both panicle and leaf blast in the nursery was further investigated and validated in a segregating population. Our analysis also confirmed that the *qPBR1* locus does not negatively affect rice yield. Furthermore, by combining transcriptomic data with GWAS results, we were able to pinpoint six candidate genes within the *qPBR1* interval. These findings present new QTLs and potential resistant genes that could be used in the molecular breeding of rice to enhance resistance against both panicle and leaf blast. This study offers new insights into the underlying mechanics of panicle and leaf blast resistance.

## Results

### Variation of Blast Resistance in an International Diverse Panel

**Fig. 1:**
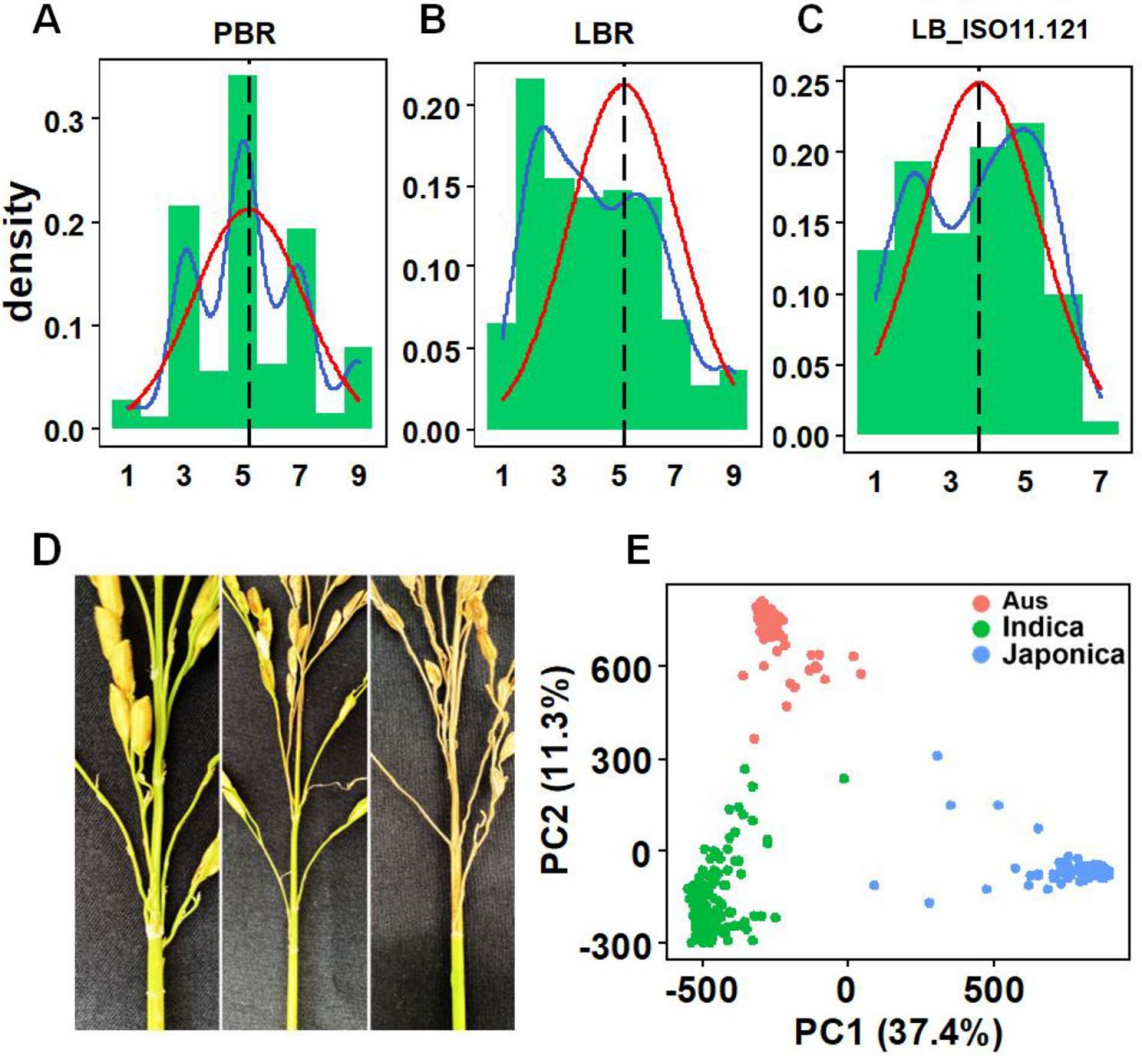
Phenotype variation and population structure. **A–C** Histogram of panicle, leaf and seedling blast resistance. Blue line: Trendline, Red line: Normal distribution line, Black line: Mean of phenotype. **D** The phenotype of representative varieties with different panicle blast resistance. **E** PCA of the rice population based SNP genotype

A panel of 414 diverse rice accessions were selected from an international collection consisting of 1,568 accessions (McCouch et al. 2016) based on their genetic diversity and representativeness, which encompasses three major subpopulations of Asian rice: *Indica*, *Japonica*, and *Aus*. To identify the broad-spectrum blast resistant loci, both panicle blast resistance (PBR) and leaf blast resistance (LBR) in the field were assessed in a blast nursery in Yangjiang, Guangdong Province, China. Leaf blast resistance of this panel to 7 isolates of *M. oryzae* (LB_ISO11.121, LB_ISO08T19, LB_ISO11.882, LB_ISO11.1093, LB_ISO13.227, LB_ISO14.42, LB_ISO98.288) at seedling stage were also accessed in a greenhouse setting. Wide variations in PBR, LBR, as well as resistance to the seven isolates were observed across the whole panel, ranging from 1 to 9, 1–9, 1–7, 1-8.5, 1–8, 1–9, 1–7, 1–8 and 1–8, averaging 5.2, 4.4, 3.8, 4.0, 4.2, 4.0, 3.3, 3.9 and 4.0 (Fig. 1, Fig. S1, Table S1), respectively. Among the population, fifteen rice accessions showed extreme blast resistance in the field (Table 1, Table S2), with the majority being the *Indica* accessions (14/15). To explore whether the 7 isolates represent a wide spectrum of the rice blast pathogen, we conducted correlation analysis between field LBR phenotypes and the 7 isolates of *M.oryzae* (Fig. S2, Table S3), There is a very weak correlation (*r* = 0.09) between LB_ISO08T19 and LB_ISO13.227. And 5 small correlations (*r* < 0.25) and 13 moderate correlations (0.25 < *r* < 0.6) were present. The mean, median, minimum and maximum of the 21 correlations were 0.46, 0.53, 0.09 and 0.77, respectively. This suggests that different rice accessions within the population exhibit varying levels of resistance against the 7 strains. Therefore, these 7 strains do not belong to a narrow spectrum. It implied the 7 isolates of *M. oryzae* used in this study represent a wide spectrum of the rice blast pathogen.

**Table 1:** Rice accessions with strong blast resistance

Accession ID	Accession name	Origin	Subpopulation	PBR	LBR
IRRI2K_554	KAUKHMWE	Myanmar	*indica*	1.0	2.5
IRRI2K_587	MAURITIUS LOCAL	Mauritius	*indica*	1.0	2.0
IRRI2K_495	ELWEE	Sri Lanka	*indica*	1.1	1.5
IRRI2K_463	CAMPONI SML	Surinam	*indica*	1.1	2.0
IRRI2K_616	PERLA	Cuba	*indica*	2.5	2.0
IRRI2K_786	ARC 18,294	India	*indica*	2.5	1.0
IRRI2K_441	B 6144 F-MR-6	Indonesia	*indica*	2.8	1.0
IRRI2K_609	NS 113	Madagascar	*indica*	2.8	2.0
IRRI2K_542	JUMA 51	Dominican Republic	*indica*	2.8	2.5
IRRI2K_509	HABIGONJ BORO 6	Bangladesh	*japonica*	2.9	1.5
IRRI2K_527	IR 43	Philippines	*indica*	2.9	2.0
IRRI2K_476	CR 5272	Costa Rica	*indica*	2.9	2.0
IRRI2K_557	KHAO DAW TAI	Thailand	*indica*	2.9	2.0
IRRI2K_634	RAY NABJA	Bhutan	*indica*	2.9	2.0
IRRI2K_453	BG 301	Sri Lanka	*indica*	2.9	1.0

### Identification and Mapping of QTLs for Rice Blast Resistance by Pan-GWAS

All 414 accessions have been sequenced with an average depth of 50X. The sequencing data was mapped to our previous rice pangenome (Wang et al. 2023) to identify pangenome-wide SNPs and “presence or absence variations” (PAV). By filtering the 15% missing data and minor allele frequency (MAF) less than 5%, a total of 5,203,416 high-quality SNPs and 4,521,593 PAVs were selected for following Pan-GWAS analysis. Both Principal Component Analysis (PCA) and phylogenetic analysis (Fig. 1E, S3, S4) revealed that the 414 accessions can be clustered into three major groups, corresponding to 208 *Indica*, 66 *Aus* and 140 *Japonica* accessions (Table S1). The blast resistance comparisons among different groups revealed no difference between *Indica* and *Japonica* subpopulations in LBR, LB_ISO11.121 and LB_ISO08T19 analysis. However, the *Indica* accessions exhibited lower leaf blast resistance but higher panicle blast resistance compared to *Japonica* (*p* < 0.01) in LB_ISO11.882, LB_ISO11.1093, LB_ISO13.227, LB_ISO14.42 and LB_ISO98.288 analysis (Fig. S5).

**Fig. 2:**
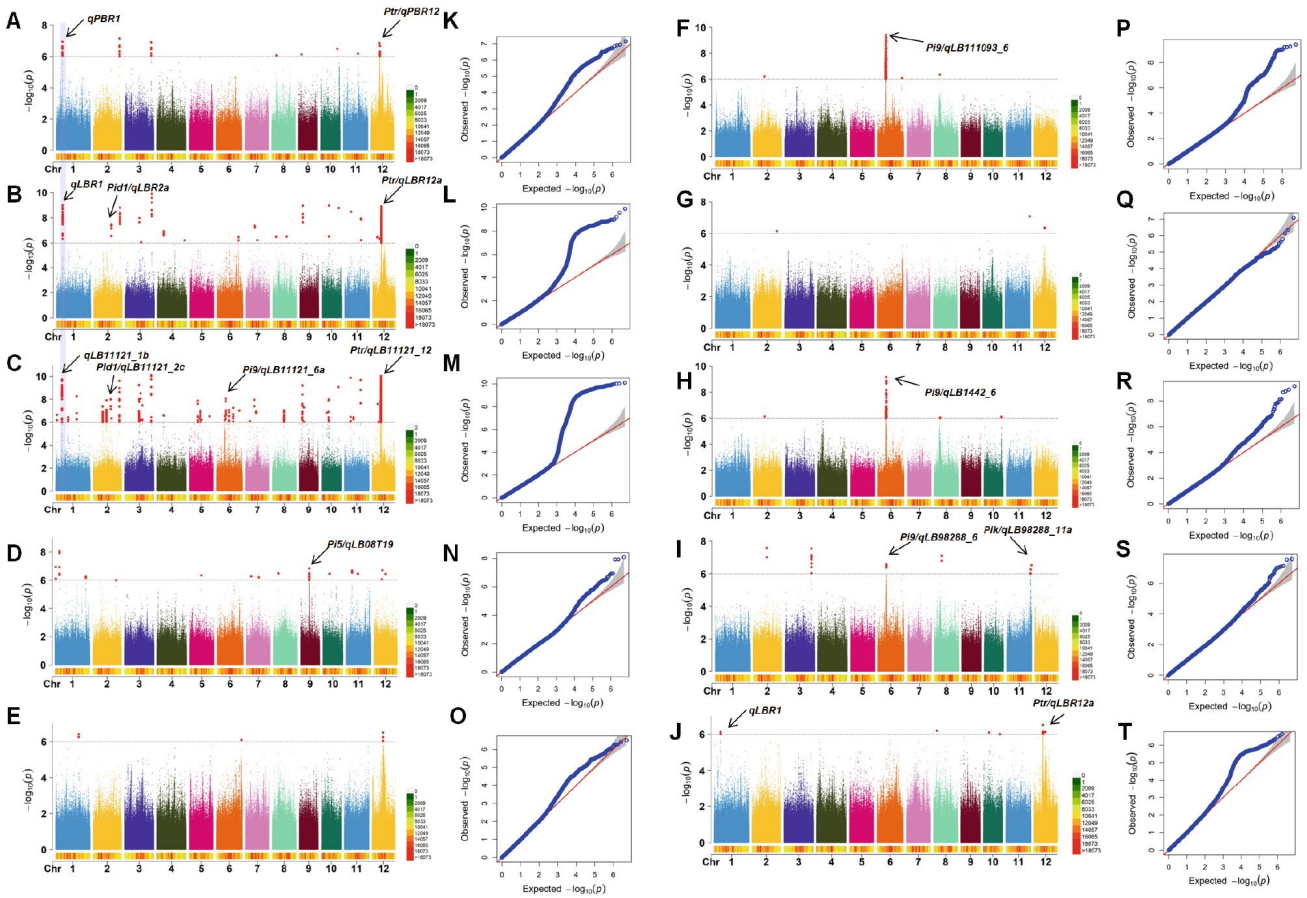
Identification of QTLs for panicle, leaf and seedling blast resistance. **A** Manhattan plots of the GWAS with panicle blast resistance (PBR) in field. **B** Manhattan plots of the GWAS with leaf blast resistance (LBR) in field with all accessions. **C–I** Manhattan plots of the GWAS with seedling blast resistance (LB_ISO11.121, LB_ISO08T19, LB_ISO11.882, LB_ISO11.1093, LB_ISO13.227, LB_ISO14.42, LB_ISO98.288) to a strain ISO11.121, ISO08T19, ISO11.882, ISO11.1093, ISO13.227, ISO14.42, ISO98.288 at seedling stage in green house. **J** Manhattan plots of the GWAS with leaf blast resistance in field without extremely resistant plants. **K–T** QQ plot for the GWAS. y-axis: observed -log10(p) and x-axis: expected -log10(p) under the assumption that p follows a uniform [0,1] distribution. The red lines and gray region show the 95% confidence interval for the QQ plot under the null hypothesis of no association between the SNP and the trait

We conducted Pan-GWAS with both SNPs and PAVs identified from our pangenome using mixed linear model (MLM) (Yu et al. 2006) in Gapit (Tang et al. 2016). The quantile–quantile (QQ) plot (Fig. 2) indicated effective control of false positives in the analysis. The linkage disequilibrium (LD) decay across the genome was analyzed in the entire panel to determine the probable genomic extent of a QTL region. In the examined population, the LD extends approximately 200 kilobases (kb) at the genomic level (Fig. S6). Consequently, we established criteria for defining QTL regions wherein any segment containing greater than two statistically significant SNPs or PAVs (*p* < 10^− 6^) within a 200 kb interval is classified as a QTL region. Both SNP-GWAS and PAV-GWAS identified QTLs for blast resistance, distributing across all 12 chromosomes and accounting for 4.5-9.7% of the phenotypic variations (Fig. 2, Fig. S7). In details, 4 (Table 2; Fig. 2aA, Fig. S7A), 18 (Table 2; Fig. 2B, Fig. S7B), 35 (Table S4, Fig. 2C, Fig. S7 C), 5 (Table S4, Fig. 2D, Fig. S7D), 3 (Table S4, Fig. 2E, Fig. S7E ), 2 (Table S4, Fig. 2F, Fig. S7F), 0 (Table S4, Fig. 2G, Fig. S7G), 1 (Table S4, Fig. 2H, Fig.S7H) and 6 (Table S4, Fig. 2I, Fig. S7I) QTLs were significantly associated with PBR, LBR and 7 strains resistance phenotypes, respectively.

Several QTLs co-localized with previously characterized blast resistant genes, confirming the reliability of our analysis (Table 2 and Table S4). For example, *qPBR12*, *qLBR12a* and *qLB11121_12* co-localized with two previously cloned genes *Pita* (Bryan et al. 2000) and *Ptr* (Zhao et al. 2018a). The *qLBR2a* and *qLB11121_2c* co-localized with *Pid1*. Additionally, the *qLB08T19_9* co-localized with *Pi5* (Lee et al. 2009), *qLB11121_6a*, *qLB111093_6* and *qLB1442_6* and *qLB98288_6* co-localized with *Pi9* (Rathour et al. 2016). The *qLB98288_11a* co-localized with *Pik* (Zhai et al. 2011) and The *qLB98288_11b* co-localized with *Pi1 (*Li et al. 2012).

**Table 2:** QTLs associated with rice blast resistance identified by GWAS

QTL	Marker	Position on NIP genome	Marker type	MAF	P-value	FDR	Phenotype contribution	Co-location gene	
**PBR**									
*qPBR1*	Chr1_9116925	8,118,681	SNP	0.22	1.15E-07	5.97E-02	5.10		
*qPBR2*	Chr2_37198859	33,002,918	SNP	0.12	7.16E-08	5.97E-02	5.27		
*qPBR3*	Chr3_37987685	32,981,449	SNP	0.32	1.24E-07	5.97E-02	5.07		
*qPBR12*	Chr12_12599463	10,798,977	SNP	0.14	2.08E-07	5.97E-02	4.88	*Ptr*	
	Chr12_12637980	10,833,939	PAV	0.22	4.45E-07	1.59E-01	4.56		
**LBR**									
*qLBR1*	Chr1_9115897	8,117,653	SNP	0.22	1.06E-09	1.72E-04	6.87		
*qLBR2a*	Chr2_24921088	21,953,715	SNP	0.19	3.34E-08	2.80E-04	5.58	*Pid1*	
*qLBR2b*	Chr2_37198859	33,002,918	SNP	0.12	5.43E-09	1.72E-04	6.26		
*qLBR3a*	Chr3_20356924	18,120,630	SNP	0.22	1.05E-08	1.78E-04	6.01		
*qLBR3b*	Chr3_37987664	32,981,428	SNP	0.22	1.24E-10	1.72E-04	7.69		
*qLBR4a*	Chr4_5270980	4,054,328	PAV	0.34	1.24E-07	1.12E-03	5.25		
*qLBR4b*	Chr4_8785383	6,618,386	SNP	0.20	1.17E-07	7.14E-04	5.12		
*qLBR4c*	Chr4_16027200	12,470,034	PAV	0.23	8.67E-07	6.23E-03	4.52		
*qLBR6a*	Chr6_27624040	23,022,166	PAV	0.12	2.32E-07	1.94E-03	5.02		
*qLBR6b*	Chr6_30626962	25,619,840	SNP	0.39	3.53E-07	1.84E-03	4.72		
*qLBR7a*	Chr7_12449656	11,050,922	SNP	0.20	4.38E-08	3.34E-04	5.49		
*qLBR7b*	Chr7_16306460	14,422,872	PAV	0.50	3.76E-08	4.04E-04	5.70		
*qLBR9*	Chr9_4512315	3,546,374	SNP	0.22	1.09E-09	1.72E-04	6.86		
*qLBR10*	Chr10_10257687	8,056,337	SNP	0.24	1.06E-09	1.72E-04	6.87		
*qLBR11a*	Chr11_1397560	1,286,775	PAV	0.21	8.74E-10	8.26E-05	7.14		
*qLBR11b*	Chr11_23163296	19,007,058	SNP	0.20	1.16E-08	1.78E-04	5.97		
*qLBR12a*	Chr12_12645340	10,841,299	PAV	0.23	5.75E-11	8.26E-05	8.21	*Ptr*	
	Chr12_12653788	10,849,747	SNP	0.22	1.23E-09	1.72E-04	6.82		
*qLBR12b*	Chr12_16714680	13,838,432	PAV	0.28	1.35E-08	1.89E-04	6.09		

PBR: panicle blast resistance QTL (GWAS with phenotypes in the field)

LBR: leaf blast resistance QTL (GWAS with phenotypes in the field)

For the newly identified QTLs, many of which can be identified in GWAS results of different phenotypes, such as *qPBR1*, *qLBR1* and *qLB11121_1b, qPBR2, qLBR2b* and *qLB11121_2d*, and *qPBR3*, *qLBR3b* and *qLB11121_3d* (Table 2, Table S4). These QTLs may be potentially QTLs involved in both panicle and leaf blast, as well as different isolate resistance.

Pangenome enables a more powerful GWAS analysis and facilitate capturing the missing heritability (Zhou et al. 2022). To confirm our pangenome-based GWAS results, we also conducted GWAS using SNPs genotyped from the Nipponbare reference genome. The comparison between GWAS from pangenome and from Nippponbare reference revealed that while most of the QTLs identified using the Nipponbare reference could also be detected in GWAS with the pangenome, our Pan-GWAS uniquely identify several novel QTLs associated with blast resistance that were not discernible using the Nipponbare reference (Fig. 2, Fig. S7, S8, Table 2, S4, S5). These results highlight that pangenome is a more powerful reference for GWAS analysis.

To reduce the affection from *R* genes in some varieties leading to qualitative resistance phenotype, we further conducted Pan-GWAS excluding accessions with extreme resistance (PBR < = 3, LBR < = 3 or LB_ISO11.121 < = 3). Notably, the *qLBR1* with the most significant SNP signal at 9,115,897 bp within the 9.01–9.21 Mb regions on chromosome 1 of the pangenome can also be identified (Fig. 2J). This suggests that *qLBR1* contributes to both qualitative and quantitative resistance to leaf blast. Furthermore, *qLBR1* also colocalized with *qPBR1*, suggesting that they might represent the same QTL conferring resistance to both panicle and leaf blast. Thus, this locus demonstrates its potential to confer not only resistance to leaf and panicle blast, but also qualitative and quantitative resistance to leaf blast.

### Validation of *qPBR1* in a Segregating Population

Both *qPBR1* and *qPBR12* have qualitative and quantitative resistance to leaf blast (Fig. 2). Particularly, the *qPBR12* had the significant SNP signal at 12,599,463 bp (10,798,977 bp on Nipponbare genome) on pangenome chromosome 12. The *qPBR12* was co-localized with a previously characterized broad-spectrum gene (*ptr*) (Zhao et al. 2018a) and contained a 42-bp deletion on the third exon of *ptr* (Fig. S9). Using a primer Z42 (Table S6) designed for the 42-bp InDel (Fig. S9), we found the 42-bp InDel significantly associated with broad-spectrum blast resistance (PBR, LBR and LB_ISO11.121) on the GWAS population (Table S7), suggesting *ptr* may be the candidate functional gene underlying *qPBR12*.

Given that the *qPBR1* locus confers resistance to both panicle and leaf blast, encompassing qualitative and quantitative resistance traits, we proceeded to rigorously validate this locus. We selected two accessions containing different haplotypes of *qPBR1* and displaying opposing phenotypes in PBR, LBR and LB_ISO11.121 from our Pan-GWAS panel for further genetic analysis (“622” as the resistant accession and “Co39” as the susceptible accession). We developed F_2:3_ population using 622 and Co39 as parents and blast resistance was accessed across the population. Phenotypic evaluation in the F_2:3_ population revealed a broad range of resistance, with an average of 4.94 for PBR and 3.46 for LBR respectively (Fig. S10, Table S8). We then created two DNA pools for bulked segregation analysis (BSA-seq). A total of 56 and 48 individuals DNA from F_2_ population with extreme phenotype in F_2:3_ population were selected for the Resistant-Bulk (scores 2–3 for PBR and 1–2 for LBR) and Sensitive-Bulk (scores 7–8 for PBR and 5–7 for LBR) respectively (Table S9).

**Fig. 3:**
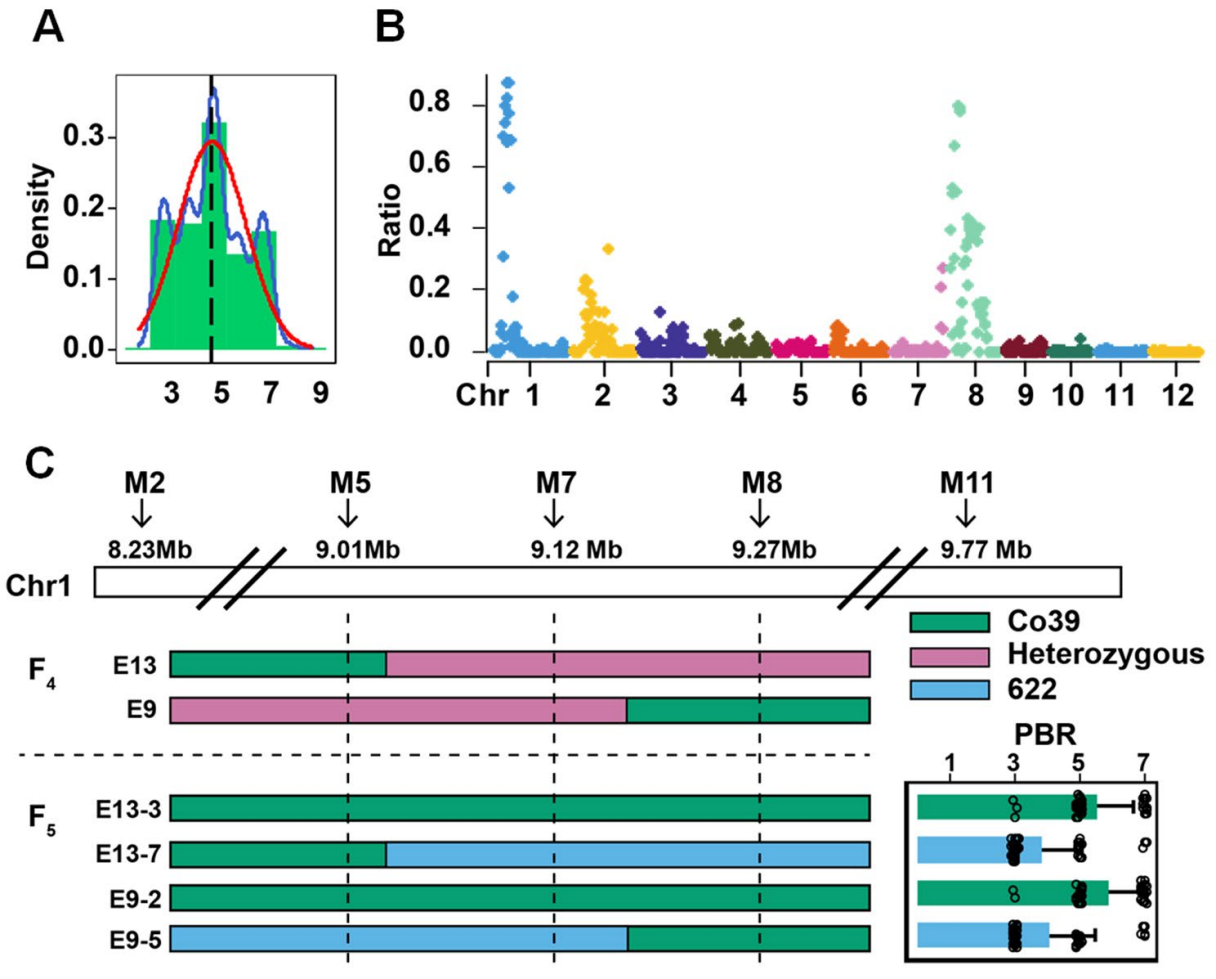
QTL mapping for panicle blast resistance (PBR) and fine mapping of qPBR1. **A** Histogram of PBR in F_4:5_ population. Blue line: Trendline, Red line: Normal distribution line, Black line: Mean of phenotype. **B** Ratio plot by GBS method. The X-axis value is set at a midpoint at each defined genomic interval and the Y-axis value corresponds to ratio. **C** Fine mapping of qPBR1-3. E13 and E9 were two heterozygous recombination plants from the F_4_ generation. The 4 F_5_ homozygous plants were derived from the 2 recombination plants. The chromosome segment represented by *green bar* indicated segment from Co39, while *red bar* indicated heterozygous, and *blue bar* indicated segment from 622. The mean PBR phenotypes of the descendant generation (F_5:6_) families represent the F_5_ homozygous individuals. *M2*, *M5*, *M7*, *M8*, *M11* were the markers for fine mapping

BSA-seq analysis revealed two genomic regions on chromosome 1 (7.4-10.2 Mb) and 8 (4.6-6.2 Mb), representing QTLs for PBR or LBR. A segment spanning 7.4-10.2 Mb on chromosome 1 co-localized with and thus validated the *qPBR1* locus. We then used a modified strategy of fine mapping (Wang et al. 2018) to narrow down the *qPBR1*. Considering the affection from the significant association on chromosome 8, we developed 2 InDel makers (Table S10) to filter the homozygous plants on chromosome 8 (4.6-6.2 Mb). Then we developed 5 InDel markers (Table S10) to filter the recombinant plants on chromosome 1 (7.4-10.2 Mb). Specifically, two recombinant plants on chromosome 1 which were homozygous on chromosome 8 were selected from the F_4_ generation and the homozygous offspring was filtered in the descendant generation (F_5_) derived from these 2 recombination plants (F_4_). The correlation tests were performed in the descendant generation (F_5:6_) derived from the homozygous F_5_ plants, revealing a significant correlation between the marker M7 (Fig. 3) and blast resistance phenotype, particularly in the F_5:6_ families from the heterozygous recombination plant E13 and E9 (Fig. 3). Upon identifying *qPBR1* as a locus conferring both substantial and durable resistance, we developed a molecular marker (Table S10) for its targeted selection in breeding programs. Fine mapping with marker M5 and M8 further narrowed down *qPBR1* to a 254 kb region on chromosome 1 (Fig. 3).

### Comparative Transcriptomic Analysis of Resistant and Sensitive Accessions

To identify functional genes underlying *qPBR1*, and dissect the molecular mechanisms of rice blast resistance, we performed a comparative transcriptomic analysis at 5-leaf stage before and after blast infection, using three resistant accessions (RAs) containing the resistant haplotype of *qPBR1*, and three sensitive accessions (SAs) containing the sensitive haplotype of *qPBR1*. To validate whether the three SAs have similar gene expression patterns, we performed a correlation analysis of the gene expression profile. Four technical duplications of expression patterns on each accession showed strong correlations of gene expression (*r* > 0.9), confirming the high-quality of the transcriptomic analysis. The strong correlations of gene expression were detected between two of the three RAs (*r* > 0.9) and three SAs (*r* > 0.85), indicating three RAs and SAs had the similar gene expression profiles. The correlation coefficient of expression patterns decreased at 12 h and increased at 48 h after inoculation, which indicated a significant shift of expression pattern at 24 h, a substantial response to the pathogen partially recovered at 48 h after inoculation (Table S11).

From sample before inoculation (0 h), we identified 1739 differentially expressed gene (DEGs) between RAs (RA_0h) and SAs (SA_0h), which included 760 up-regulated and 979 down-regulated genes. After 24 h inoculation, we identified 2125 DEGs (1222 up- and 903 down-regulated) between RAs and SAs with 24 h and after 48 h inoculation, 3148 (1359 up- and 1789 down-regulated) DEGs were detected between RAs and SAs, with the distribution patterns of DEGs shown by the scatter plot (Fig. S11).

To determine the genes responding to rice blast fungus inoculation, we compared the DEGs at 0 h with those at 24 h (or 48 h) between RAs and SAs. Venn analysis was also performed to reduce the influence caused by genetic background effects. In total, we identified 5976 up-regulated genes between control (no rice blast fungus inoculation) and following a 24-h inoculation, with 1403 and 492 up-regulated genes being unique to RAs and SAs respectively. Meanwhile, 3188 and 3576 up-regulated gene were found in RA_0-48 h and SA_0-48 h. 5114, 5369, 4248 and 4724 down-regulated genes were found in RA_0-24 h, RA_0-48 h, SA_0-24 h and SA_0-48 h, respectively. The DEGs (yellow region with red digital, in Supplementary Fig. S12) captured in the RAs between control and rice blast fungus inoculation, pinpointing potential blast resistance-related genes. Considerig the LRR (Leucine-rich repeat) genes are often implicated in plant defense mechanisms, the Venn analysis was also performed for LRR genes in the DEGs. There are 108 LRR genes in the DEGs (Table S12).

Gene ontology (GO) analysis of the DEGs (Fig. S12) was performed, with 2962 up-regulated DEGs (Fig. S12A) enriched in 24 GO terms (*p* < 0.05) (Table S13). The most significant enriched GO terms in biological process (BP) was cellular lipid metabolic process (GO:0044255). Meanwhile, the 3014 down-regulated DEGs (Fig. S12B) were enriched in 7 GO terms (Table S14).

### Candidate Gene Identification in the *qPBR1*

To identify the functional gene underlying *qPBR1*, results from transcriptomic and genetic analysis were combined to narrow down the candidate genes. There are 33 genes located in the interval of *qPBR1* (Chr1: 9,0–9,2 Mb on the pangenome; Chr1: 8.0-8.2 Mb on the Nipponbare genome), of which 23 genes showed no expression (FPKM < 1) in all analyzed samples. In addition, no coding sequence variation was found in these non-expressed genes between the accessions “Co39” and “622”, two parents of the population for validating the *qPBR1* locus.

**Fig. 4:**
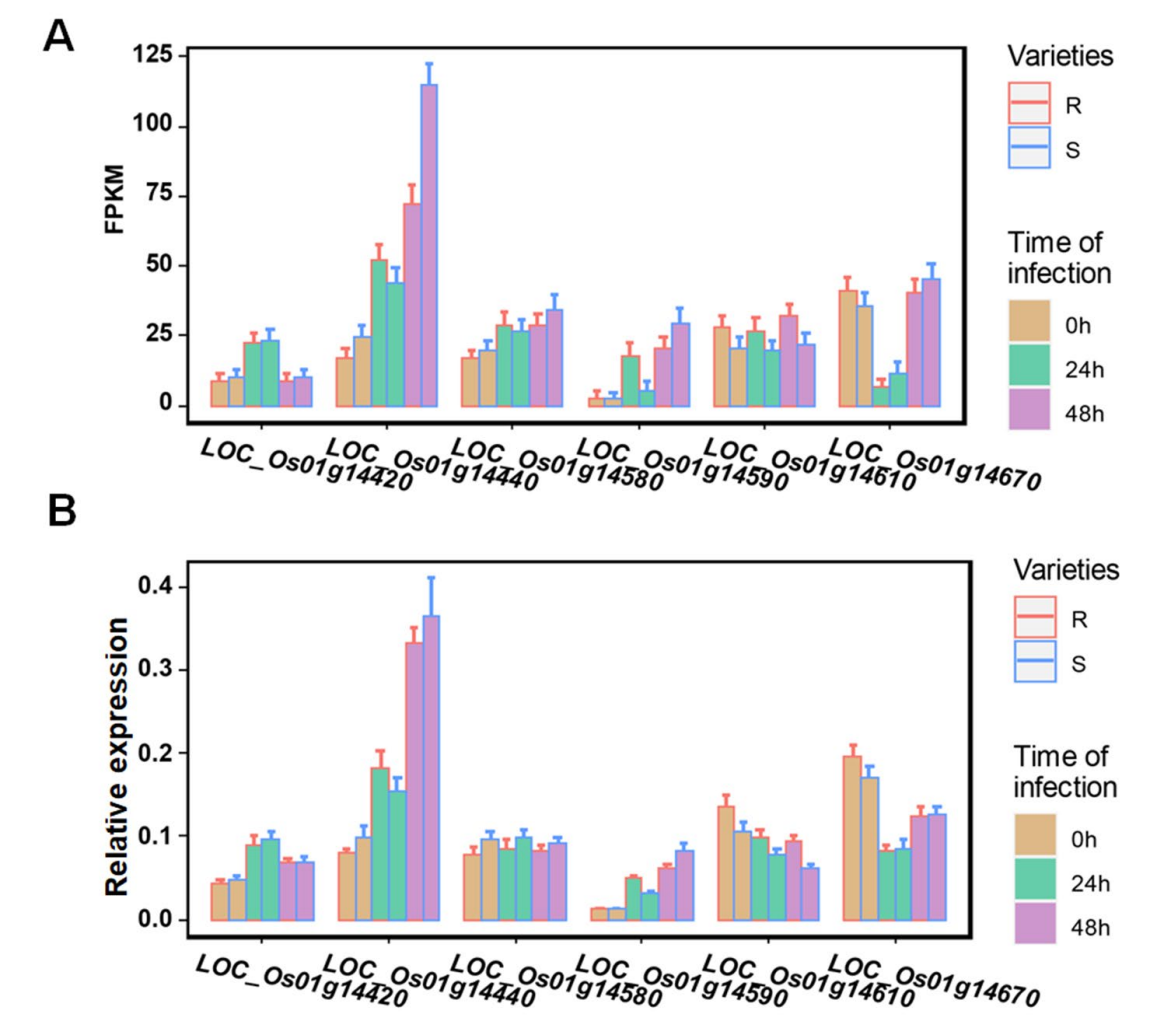
Analysis of DEGs in the qPBR1-3. **A** FPKM of the 6 DEGs between RA and SA at 0 h, 24 h and 48 h. **B** Relative expression of 6 DEGs with qRT-PCR between RA and SA at 0 h, 24 h and 48 h

Among the remaining 10 genes, four genes showed no differential expression between RAs and SAs. And no coding sequence variation was found in the coding region of the four genes between the accessions “Co39” and “622”. The remaining six genes (*LOC_Os1g14420*, *LOC_Os1g14440*, *LOC_Os1g14580*, *LOC_Os1g14590*, *LOC_Os1g14610* and *LOC_Os1g14670*) showed differential expression between RAs and SAs, or showed expression induction by blast fungus inoculation. These genes encode RWP-RK domain-containing protein, WRKY1 protein, dehydrogenase, pathogen-related protein, PSF2 - Putative GINS complex subunit and Cupin domain containing protein, respectively. The expression levels of *LOC_Os1g14420*, *LOC_Os1g14440*, *LOC_Os1g14580* and *LOC_Os1g14590* significantly increased in RAs after inoculation, while the expression levels of *LOC_Os1g14670* significantly decreased at 24 h after inoculation. Moreover, the expression levels of *LOC_Os1g14610* were significantly higher in RAs than SAs. The expression patterns of these genes were further confirmed by qRT-PCR assays (Fig. 4, Table S6). Therefore, these 6 genes were considered as the most possible candidate genes for *qPBR1*. We also analyzed the promoter sequences (2k upstream of the transcriptional start site) of these six candidate genes in the Pan-GWAS population and found one SNP in the promoter of *LOC_Os1g14580*. This SNP shows significant association with resistance in both the PBR natural population and the Heterogeneous Inbred Family (HIF) (Fig. S13).

### The *qPBR1* does not have Notable Negative Effect on Yield in Rice

**Fig. 5:**
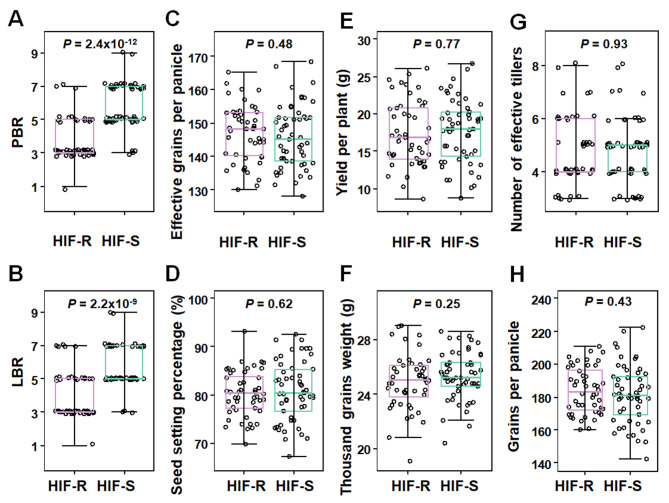
Analysis of blast resistance and yield-related traits between the individuals with blast-sensitive genotype (HIF-S) and the individuals with blast-resistant genotype (HIF-R). **A** PBR: panicle blast resistance. **B** LBR: leaf blast resistance. **C** effective grains per panicle. **D** seed setting percentage. **E** yield per plant. **F** thousand grains weight. **G** number of effective tillers. **H** Grains per panicle. *Box edges* represent the 0.25 quantile and 0.75 quantile with the median values shown by *bold lines*. ymin (*lower whisker*) = smallest observation greater than or equal to lower hinge − 1.5 * IQR (interquartile range). ymax (*upper whisker*) = largest observation less than or equal to upper hinge + 1.5 * IQR

To demonstrate the breeding value of *qPBR1* in improving rice blast resistance, we further investigated the impact of *qPBR1* on rice yield traits. A F_5_ individual heterozygous of *qPBR1* was filtered by three markers from E13 (Fig. 3C, F_4_) and the HIF derived from the F_5_ individual was genotyped by three markers. In total, 106 individuals with homozygous “Co39” chromosome fragment in the *qPBR1* (blast sensitive genotype) and 96 individuals with “622” chromosome fragment (blast resistant genotype) in the *qPBR1* were generated. Field assessments confirmed that while *qPBR1* conveyed blast resistance, it did not have notable adverse impact on yield traits (Fig. 5), making it a favorable allele for enhancing blast resistance in rice without yield penalties.

## Discussion

### Pan-GWAS is a Powerful Method for Dissecting the Rice Blast Resistance

GWAS using a globally diverse germplasm and a pangenome reference is a robust strategy for dissecting the genetic basis of important traits in rice. Rice blast disease is a major, recurrent threat to rice cultivation worldwide, and while host resistance is the most effective and economic defense, it often breaks down due to the genetic instability in cultivated rice and the pathogen’s variability. Hence, it is urgent to characterize more blast resistant QTLs from the diverse germplasms. The germplasms used in this study, representing global diversity, provided a solid foundation for identifying multiple resistance mechanisms and successfully pinpointing 15 exceptional resistant accessions. These resistant germplasms are ideal for breeding programs for improving blast resistance in rice. Furthermore, sequencing all 414 accessions at a high depth ensured accurate genotyping and comprehensive GWAS analysis in the present study.

Pangenomes represent the complete genetic diversity within a species (Tettelin et al. 2005), capturing the genetic variations absent in a single reference genome due to structural variations (SVs) among individuals (Bayer et al. 2020; Hu et al. 2024; Zhao et al. 2020). In this study, the pangenome was used as a reference genome to conduct GWAS to identify QTL conferring blast resistance, yielding a higher mapping rate than the Nipponbare reference and enabling more accurate GWAS results free from reference bias. This is in line with our previous findings (Wang et al. 2023). Additionally, we found that Pan-GWAS using PAV as markers not only confirmed QTLs identified by Pan-GWAS using SNP, but also unveiled novel QTLs, underscoring the value of using PAV as complementary markers for GWAS. Comparisons between GWAS results using the pangenome and the Nipponbare genome revealed a more comprehensive QTL detection with the pangenome, encompassing almost all QTLs identified using the Nipponbare genome. Furthermore, some phenotypically associated SNPs or PAVs uniquely detected by the Pan-GWAS often lay in regions absent in the Nipponbare genome, affirming the advantages of pangenome-based genotyping in QTL detection. Taken together, our collective assortment of international diverse germplasms, as well as using pangenome for GWAS analysis, offered a promising method for exploring and dissecting the genetic foundation of rice blast resistance.

### Identification of Major and Broad-Spectrum Rice Blast Resistant QTLs

It has been proven that there exists multiple diverse mechanisms underlying rice blast resistance. We conducted Pan-GWAS analysis specifically targeting the evaluation of panicle and seedling blast resistance in a nursery. This aimed to identify QTLs exhibiting broad-spectrum resistance by taking into account the diverse fungal isolates encountered in the field. At present, most of the studies on rice blast disease resistance focused on seedling blast, with limited studies on panicle blast. However, the yield reduction inflicted by panicle blast infection is twice as severe as leaf blast, with losses approaching up to 70% of the anticipated yield under epidemic conditions (Puri et al. 2009). Therefore, rice cultivars with leaf blast resistance but without panicle blast resistance can still lead to great yield losses. It is essential to identify major and broad-spectrum blast-resistant QTLs/genes. In this study, our Pan-GWAS results identified many leaf and panicle blast-specific QTLs, suggesting distinct resistance mechanisms underlying leaf and panicle blast, and underscoring the necessity of integrating molecular marker-assisted breeding to achieve a balance for effective resistance to both panicle and leaf blast. The QTL allowing major and broad-spectrum resistance is an ideal candidate for improving rice leaf and panicle blast resistance through breeding.

Blast resistance can be classified into complete or partial resistance (Wang et al. 1994). The complete resistance is qualitative (highly efficient) and race specific, involving major *R* genes such as *Pib* (Wang et al. 1999), *Pita* (Bryan et al. 2000), and *Pi9* (Qu et al. 2006). The partial resistance has minor effect and lower efficiency to defend against *M.oryzae*, involving resistance genes such as *pi-21* (Fukuoka et al. 2009), while offering durability to different *M.oryzae* isolates due to its non-race-specific and polygenic features. By combining complete and partial resistance genes, we aim to counteract the transitory nature of blast resistance in rice cultivars. By excluding accessions with extreme resistance (PBR < = 3, LBR < = 3 or LB_ISO11.121 < = 3), we try to eliminate the complete resistance conferring by major *R* gene. By this way, our GWAS results demonstrated that *qPBR1* and *qPBR12* may be QTLs conferring quantitative resistance to both leaf and panicle blast (Fig. 2D). Since *ptr* (Zhao et al. 2018a) may be the candidate functional gene underlying *qPBR12*, the gene act as a QTL with partial resistance.

From the GWAS results, we found that *qPBR1* and *qLB11121_1b* were in the same locus, implying they may be the same QTL. Isolate ISO11.121 is a prevalent race commonly found in rice fields and can represent partial field population of *M. oryzae* in South China (Xiao et al. 2020). By inoculating with ISO11.121, it is theoretically possible to screen rice varieties for broad-spectrum resistance that should effectively counter a wide range of races represented by ISO11.121. While the resistance level of these varieties might not be as extensive as that offered by *Pi9* - which was identified in GWAS involving three strains (*qLB111093_6*, *qLB1442_6*, *qLB98288_6*) - they are still considered to have significant broad-spectrum resistance (Qu et al. 2006; Rathour et al. 2016). In the rice field, there are theoretically infinite races of rice blast fungus theoretically. It suggests that *qPBR1* is a broad-spectrum resistance gene. Moreover, *qPBR1* emerges as a novel QTL offering both complete and partial resistance without adversely affecting yield. This makes *qPBR1* an excellent candidate for breeding new, durable, blast-resistant rice varieties.

### Identification of Candidate Genes Underlying *qPBR1*

Our transcriptomic profiling of three blast-resistant and three blast-sensitive accessions after inoculation has provided novel insights into the genes potentially involved in blast resistance. Furthermore, by comparing gene expression in the rice accessions at different time points after inoculation, we successfully identified functional genes induced by *M. oryzae*, which mediated immune response or are involved in *M. oryzae* inoculation. Merging these DEGs with other genetic analyses conducted in the present study, including Pan-GWAS and gene structure assessment, aided in fine-mapping *qPBR1* to a 200 kb region then identifying six candidate genes in this locus. This significantly contributed to our understanding and further characterization of the genetic base of *qPBR1*.

Given that *qPBR1* may provide both qualitative and quantitative resistance according to our results, we hypothesized the presence of a major *R*-gene(s) with NLR features in the linked regions of *qPBR1*. However, none of the 33 annotated genes in this region were classified as typical NLR genes. This suggests the functional gene underlying *qPBR1* may be the atypical resistance gene which is mediated by the NLR *R* genes. The six candidate genes were identified based on gene expression patterns and the defined QTL region. We examined the promoter regions, extending to 2 kb upstream, of these six genes and compared the sequences between susceptible line Co39 and resistant line 622, revealing one SNP in the promoter of *LOC_Os1g14580*. Further haplotype analysis and statistical t-tests revealed a significant association of this SNP with panicle blast resistance in both the natural population and the Heterogeneous Inbred Family (Fig. S13), indicating *LOC_Os1g14580* is the most likely candidate gene for *qPBR1*. Further transgenic experiments are necessary to definitively identify the functional genes responsible for *qPBR1*.

## Conclusion

By combining the Pan-GWAS and transcriptomic analysis, this study provided a collection of QTLs and DEGs pivotal for understanding rice panicle and leaf blast resistance. A novel QTL (*qPBR1*) showing broad-spectrum resistance to both leaf and panicle blast and six candidate genes were successfully identified in this locus. Future studies will focus on identifying the functional gene underlying *qPBR1* and incorporating this QTL/gene into breeding programs for new rice varieties with comprehensive and lasting blast resistance. This study provided valuable QTLs and candidate genes for future improving rice sustainable panicle and leaf blast resistance by molecular breeding.

## Methods

### Plant Materials

From an international panel with 1568 rice accessions (McCouch et al. 2016), 414 genetically diverse rice accessions from 56 countries were selected. Seeds were sourced from the International Rice Research Institute (IRRI). Additionally, an F_2:3_ population consisting of 503 individuals was developed from a cross between the blast-susceptible *Indica* rice accession “Co39” (IRRI2K_19) and the resistant *Indica* accession “622” (IRRI2K_622, “PINURSIGI”). The Heterogeneous Inbred Families derived from “Co39” and “622” were planted in the normal paddy field environment and blast nursery. We investigated yield-related traits of the homozygous individuals without blast infection conditions.

### Evaluation of Rice Blast Resistance

Both the GWAS population and the F_2:3_ population were planted at the experimental field of Yangjiang Institute of Agricultural Sciences (111.840994°E, 21.879502°N), an area known for natural occurrence of rice blast disease. Leaf blast response (LBR) was assessed during heading stages and panicle blast (PBR) was recorded at maturation stages, with each population undergoing three biological replications. For each replication, 16 plants per accession family were planted into two rows, and the middle 12 plants were scored for blast resistance. The median response of each family was used as the effective phenotype for one replication and the mean phenotypes of three replications was used for GWAS analysis. To ensure the data reflect the same development stages, the plants which developed asynchronously at heading stage were excluded. The most seriously diseased leaf of the top two or three new leaves was scored for each plant using the rating scale, where 1 = no evidence of infection or brown specks smaller than 0.5 mm in diameter, no sporulation; 3 = brown specks with 0.5–1.0 mm in diameter, no sporulation; 5 = roundish to elliptical lesions, 1–3 mm in diameter, grey center surrounded by brown margins, lesions capable of sporulation; 7 = typical spindle-shaped blast lesions capable of sporulation, 3 mm or longer; 9 = lesions as in 7 but about half of one to two leaf blades killed by coalescence of lesions. Panicle blast severity was estimated as a percentage of infection on the rice panicle neck at maturation stage. The percentage of infection was evaluated by calculating the percentage of infected main axis length (Percent infected main axis length = infected main axis length/main axis length of the inoculated panicle). We still use the rating scale method for panicle blast resistance based on the percentage of infection. For evaluation of the leaf blast response to 7 *M. oryzae* isolates at seedling stages, the rice plants of GWAS population were grown in a greenhouse. The seeds were sowed in a plastic tray with sterilized soils from the field. One tray was divided into 24 line areas. Each line contained 8 plants. The seedlings were grown in the greenhouse at 24 to 30 °C with 8 h dark and 16 h light cycle. At V4 stages, the tray would be placed in large black iron box for inoculation with a spore suspension of *M. oryzae* isolates. Plants were inoculated with 25 mL of the filtered *M. oryzae* isolates spore suspensions by an airbrush and placed inside the large black sealed iron box for 48 h at 21 to 24 °C. After that, the inoculated plants were transferred to a greenhouse with an 8 h light and 16 h dark cycle with 80% relative humidity for additional 7 days. Disease reactions were evaluated on the second youngest leaf, using a categorical rating system from 1 (resistant) to 9 (susceptible), where 1 = no visible lesion or a few small point lesions; 3 = lesion size smaller than 2 mm without obvious fungal mass; 5 = 10% of leaf area with lesions bigger than 2 mm; 7 = 10–50% of the leaf with lesions bigger than 3 mm; 9 = more than 50% of leaf area with lesions bigger than 3 mm. The disease reactions had three biological replications and each line of one replication contained 8 seedlings. The mean disease reaction value was used for analysis.

### Genome-Wide Association Study

DNA was extracted from fresh leaf tissue using the CTAB (hexadecyltrimethylammonium bromide) protocol. Sequencing of each DNA sample was performed on the Illumina NovaSeq6000 platform and the sequencing data were aligned to a published rice pangenome (Wang et al. 2023) with BWA-MEM (Li 2013). Nucleotide variants were called using GATK (McKenna et al. 2010). The principal component analysis was performed with Gapit version 2 (Tang et al. 2016) pipeline. LD decay analysis was performed with PopLDdecay v3.40 (Zhang et al. 2019). The phylogenetic tree of the population was performed by SNPhylo v20180901 (Lee et al. 2014). GWAS was conducted using Gapit version 2 (Tang et al. 2016) with a mixed linear model. Manhattan plots were generated using the R package ggplot2 (Wickham, 2016).

### BSA QTL Mapping

Two bulks were separated from the F_2:3_ population based on the rice blast resistance phenotype. Fifty-six individual DNAs from F_2_ population exhibiting extremely resistant phenotypes were pooled to form Resistant-Bulk and another 48 individual DNAs showing extremely sensitive phenotypes were combined to create the Sensitive-Bulk. All DNAs in each bulk were properly mixed with equal concentration. The four DNA pools (“Co39”, “622”, Resistant-Bulk and Sensitive-Bulk) were sequenced using the Illumina NovaSeq6000 platform and the sequencing data were aligned to rice pangenome with BWA-MEM (Li 2013) and nucleotide variants were called using GATK (Tang et al. 2016).

### RNA-Sequencing and Gene Differentiation Expression Analysis

We classified rice accessions with a PBR ranking of 1–3 and a LBR ranking of 1–2 as Resistant Accessions (RAs), while those with a PBR ranking of 7–9 and an LBR ranking of 5–9 were categorized as Sensitive Accessions (SA). We chose three RAs (“622”, IRRI2K_561, IRRI2K_453) and three SAs (“Co39”, IRRI2K_667, IRRI2K_1354) from the GWAS population randomly for transcriptome analysis. All samples were sown in a plastic tray with sterilized soils at the greenhouse. At V4 stages, the seedling was placed in a large black iron box for inoculation with a spore suspension of *M. oryzae* ISO11.121 isolates. At 0 h, 24 h, and 48 h after inoculating, four biological RNA replicates of one sample were extracted with the RNeasy Kit (AiDeLai, China) from the seedling (without root). The RNA samples were evaluated on agarose gels, quantified in a spectrophotometer, and then stored at -80°C. The RNA samples were then sequenced using a HiSeq-2500 instrument, and 10 Gb of raw sequencing data were produced. Trimmomatic version 0.33 (Bolger et al. 2014) was used for removing the raw RNA-seq read adapter sequences and low-quality bases in the paired-end mode with recommended parameters. The virus-like and rRNA-like RNA-seq reads were further removed with fastq_clean (zhang et al. 2014). Finally, the clean data were mapped to the reference genome (MSU V7.0) with STAR (Dobin and Gingeras 2015) version 2.5.0b with the parameters “–runMode alignReads–twopassMode Basic–outSAMstrandField intronMotif–outFilterMultimapNmax 1–genomeDir GenomeIndex–sjdbGTFfile Msu70.gft–alignIntronMax 30,000–sjdbOverhang 100–outSAMattributes All–outSAMattrIHstart 0–outSAMtype BAM SortedByCoordinate–quantMode GeneCounts”. The outSAMattrIHstart parameter was changed to 0 for compatibility with downstream software Cufflinks. Gene expression levels were detected by Cufflinks and cuffdiff2 (Trapnell et al. 2013) with the parameters “-library-norm-method classic–fpkm–emit-count-tables–L label1,label2 Msu70.gtf sample1.rep1.cxb, sample1.rep2.cxbsample2.rep1.cxb, and sample2.rep2.cxb’. Fragments per kilo-base of exon per million fragments mapped (FPKM) were used for describing gene expression. Genes with low expression values (FPKM < 1) were filtered for downstream analysis. Genes that were differentially expressed between the three RAs and three SAs were identified based on their corrected p-values. Gene ontology (GO) analysis was performed through R/AnnotationHub (https://bioconductor.org/packages/release/bioc/vignettes/AnnotationHub/inst/doc/AnnotationHub.html).

### Differential Expression Analysis of Genes by qRT-PCR

First-strand cDNA was synthesized from RNA using the PrimeScript ^TM^ RT reagent kit (TaKaRa, Japan). The house-keeping *ubiquition* gene was used as an internal control. Real-time PCR was carried out using the SYBR Premix ExTaq TM kit (TaKaRa, Japan) on a Bio-Rad CFX 96 Real-Time System. All reactions were run in triplicate. Relative expression levels were calculated with the 2^−△△CT^ method (Livak and Schmittgen 2001).

### Data Analysis

Student’s t-test was used to test the significance of difference.

### Electronic Supplementary Material

Below is the link to the electronic supplementary material.

Supplementary Material 1

Supplementary Material 2

## Data Availability

No datasets were generated or analysed during the current study.
