# Supplementary material for: Pangenome-Wide Association Study and Transcriptome Analysis Reveal a Novel QTL and Candidate Genes Controlling both Panicle and Leaf Blast Resistance in Rice
Source: Rice (N Y). 2024 Apr 12;17:27. doi: 10.1186/s12284-024-00707-x (PMC11014823; doi:10.1186/s12284-024-00707-x)
Supplement: Supplementary file 2 — Supplementary Material 2 [file 12284_2024_707_MOESM2_ESM.docx]

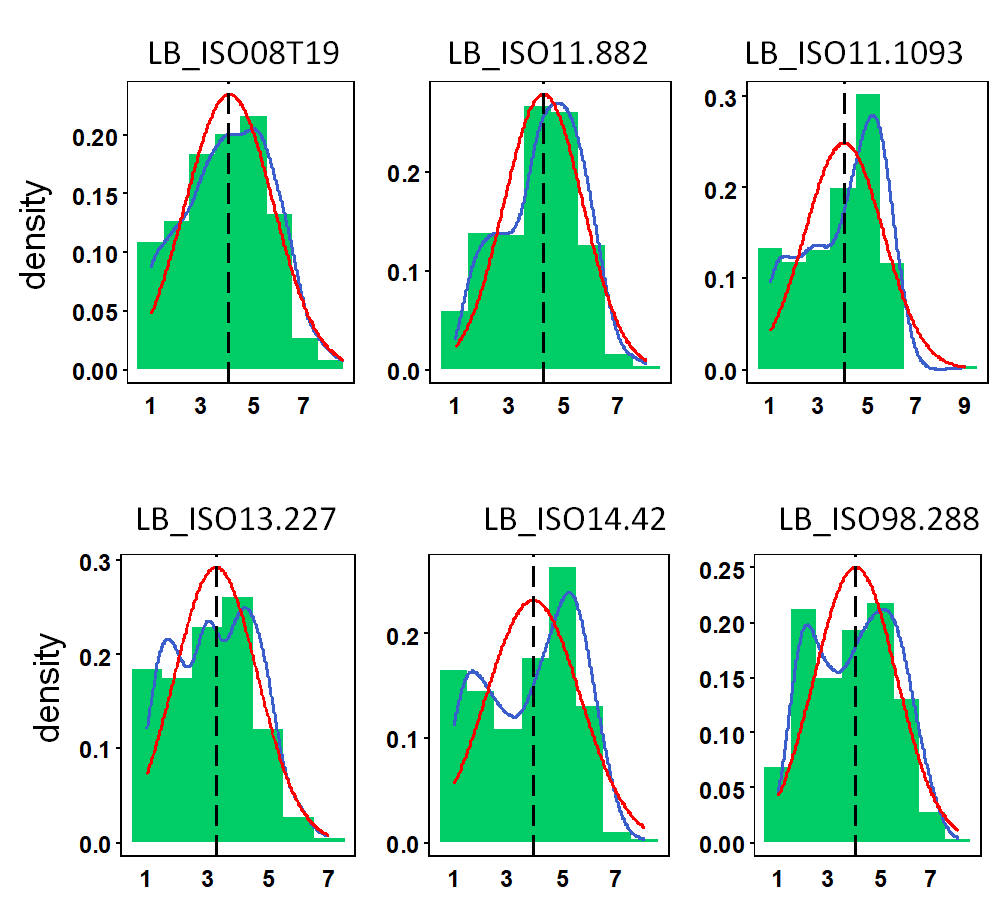


**Figure S1** Histograms of phenotype variations for 6 strains. Blue line: Trendline, Red line: Normal distribution line, Black line: Mean of phenotype.


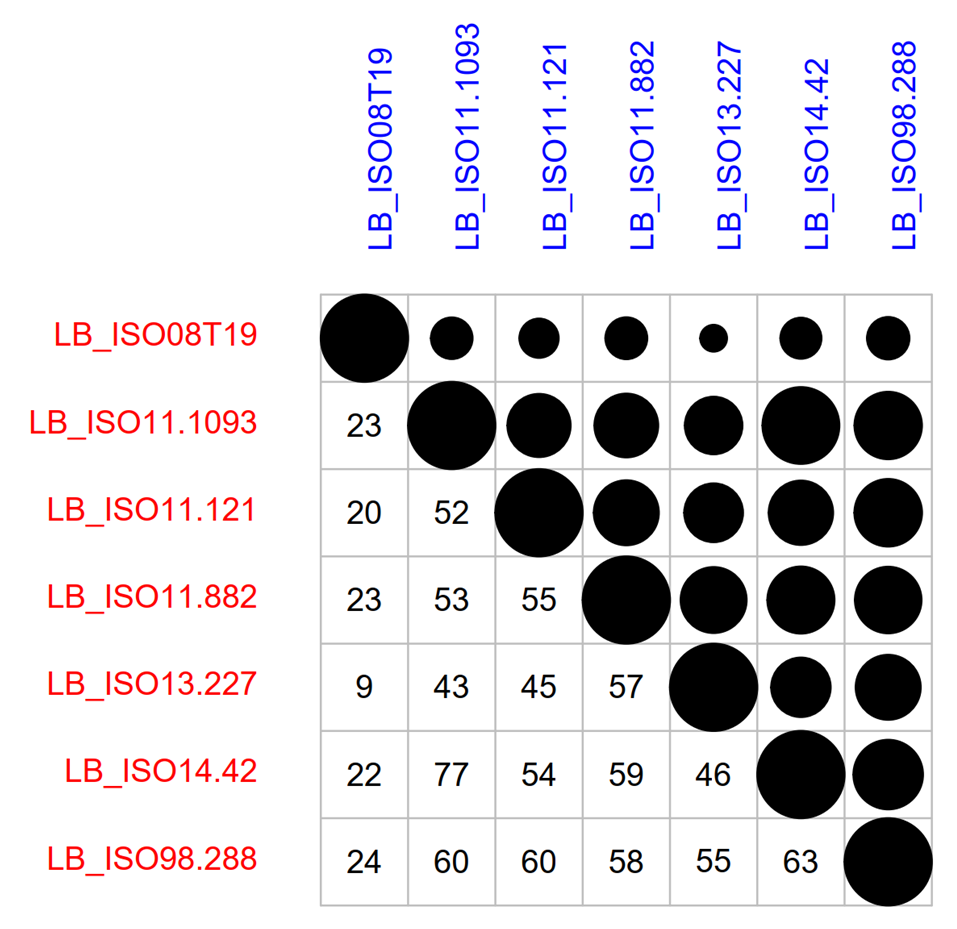


**Figure S2** Correlation analysis of phenotypes of leaf blast resistance to 7 isolates of *M. oryzae*


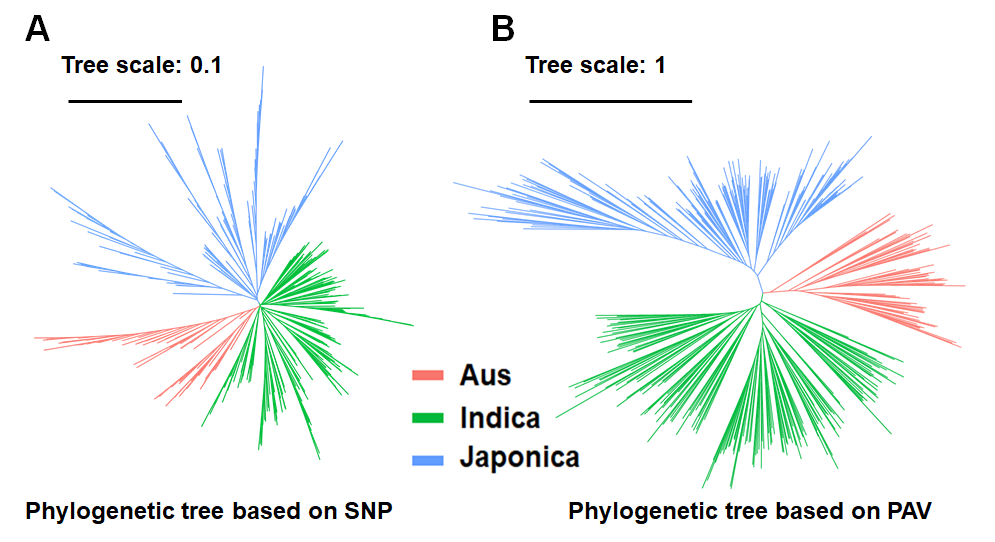


**Figure S3** Phylogenetic tree of the population


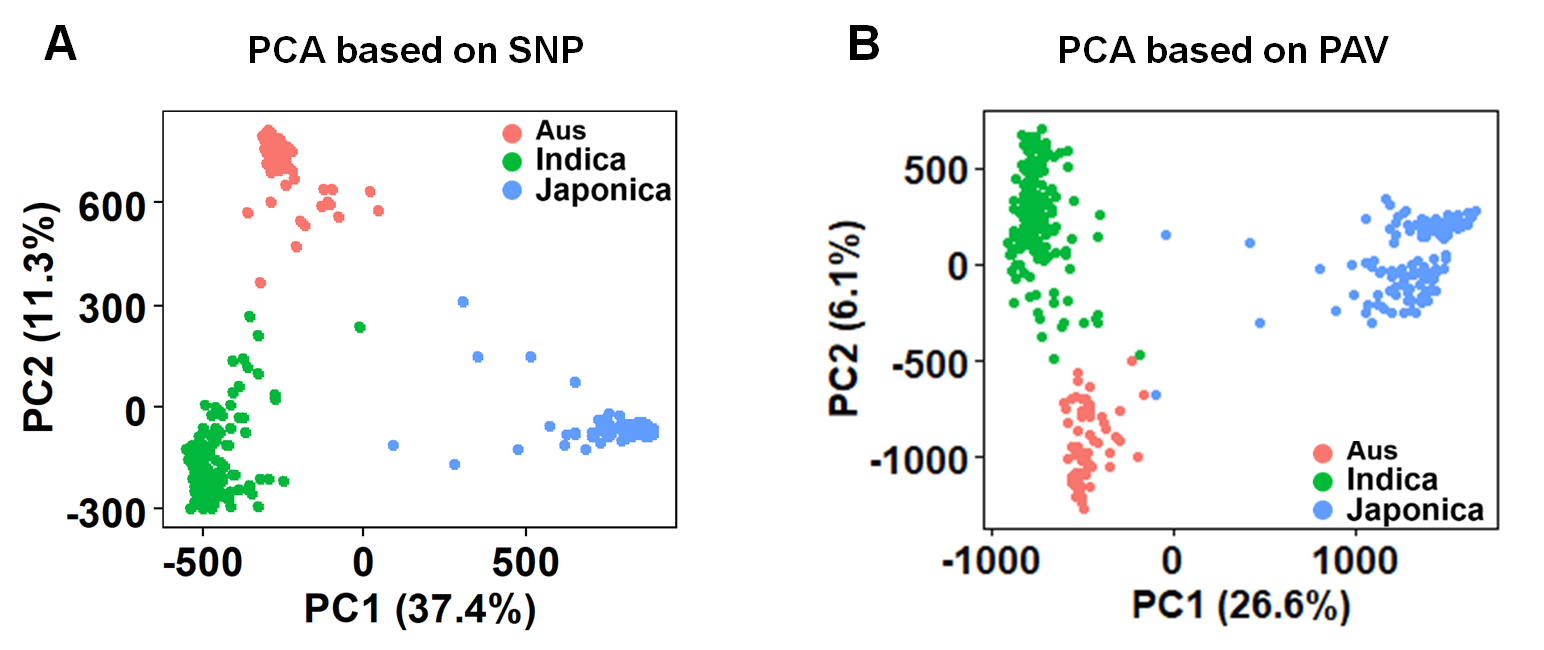


Figure S4 PCA of the population.


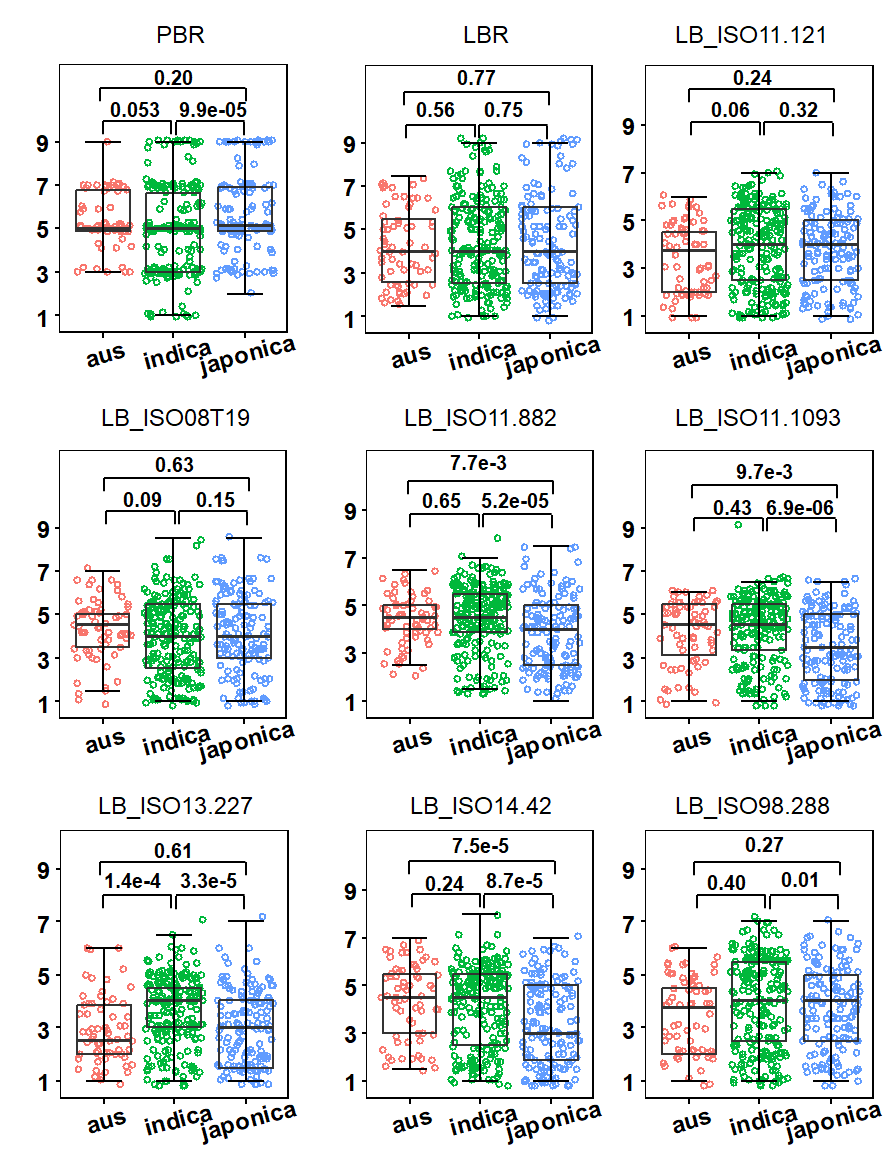


**Figure S5** Blast resistance variation in three subpopulations for panicle blast resistance (PBR) in field, leaf blast resistance (LBR) in field, and leaf blast resistance (LB_ISO11.121, LB_ISO08T19, LB_ISO11.882, LB_ISO11.1093, LB_ISO13.227, LB_ISO14.42, LB_ISO98.288) to 7 strains at seedling stage in green house


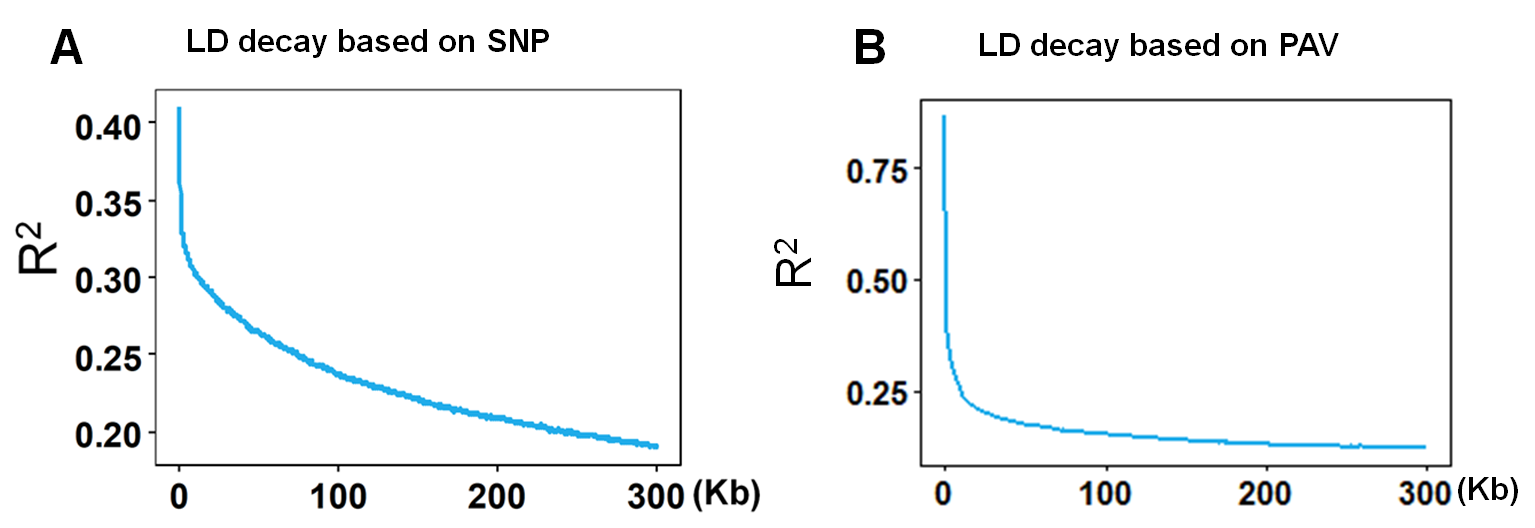


**Figure S6** Genome-wide average LD decay estimated in 414 rice accessions


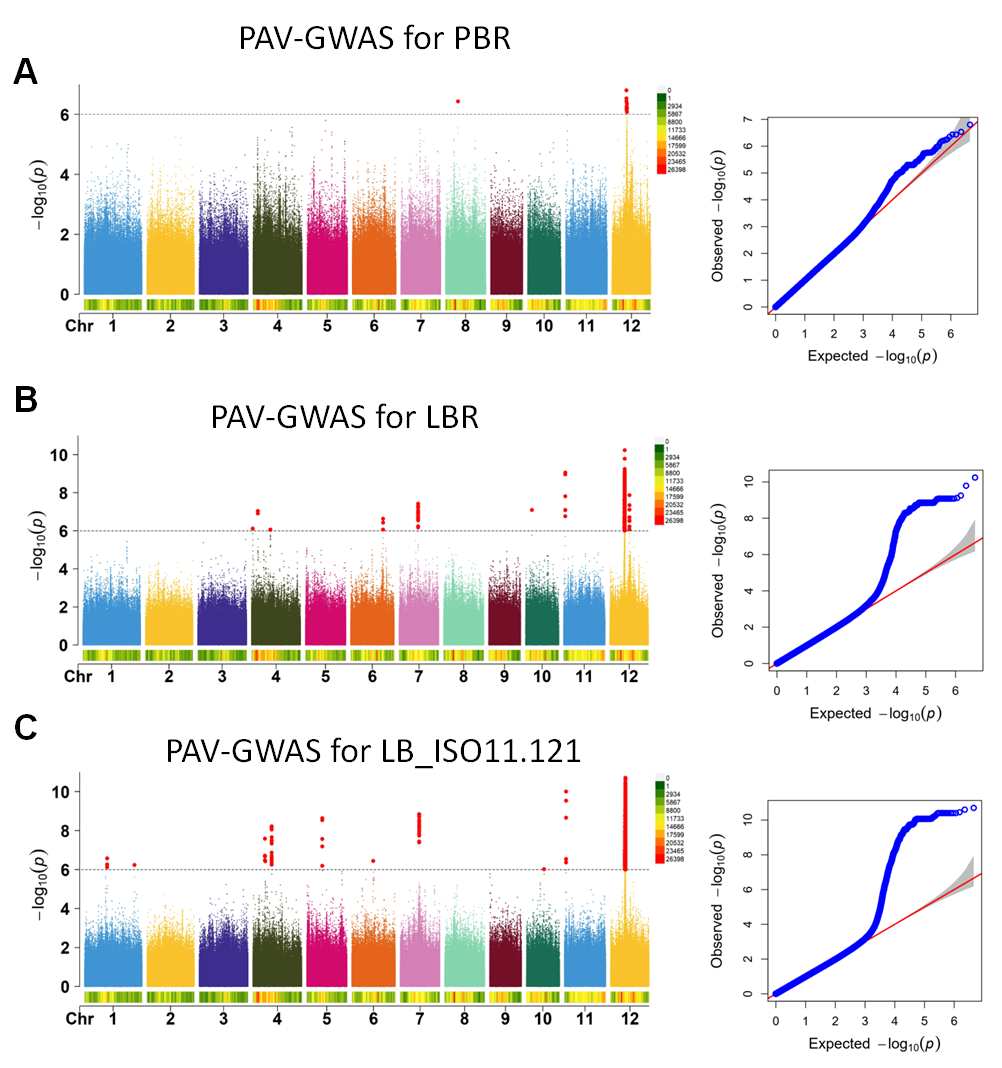

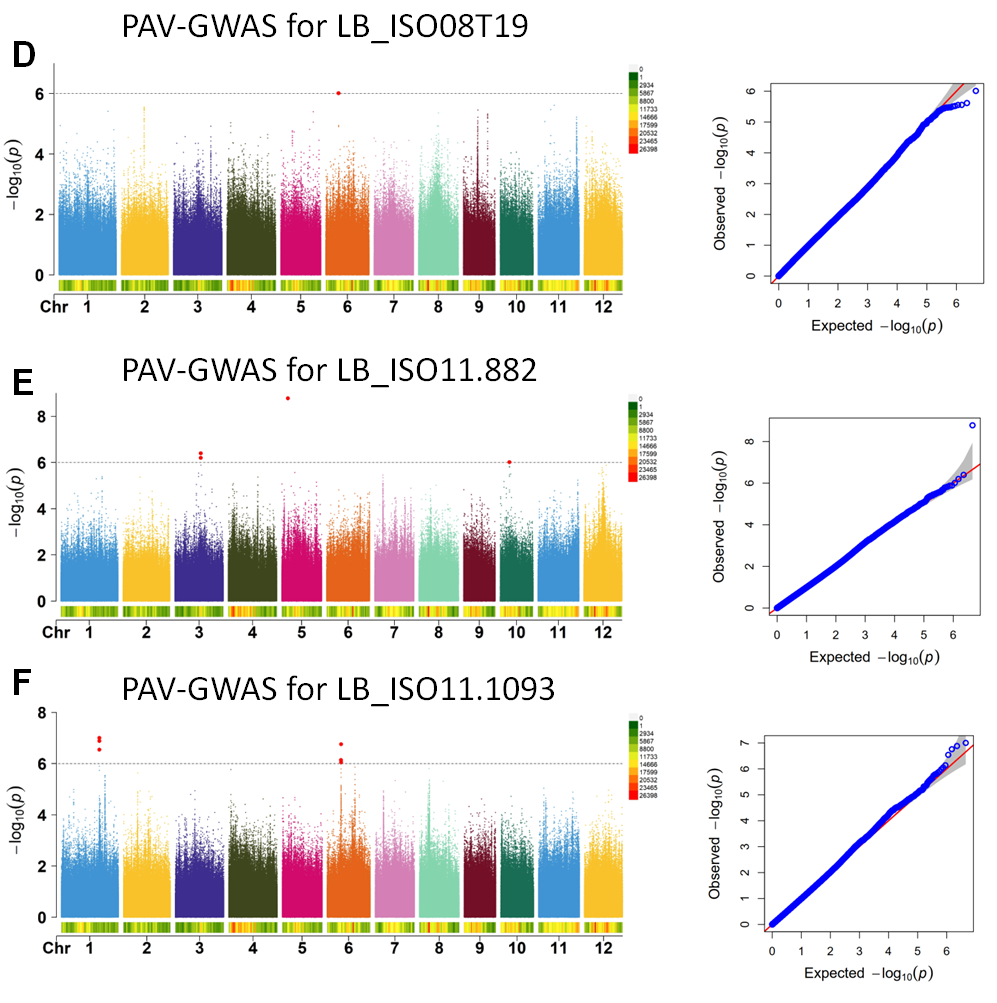

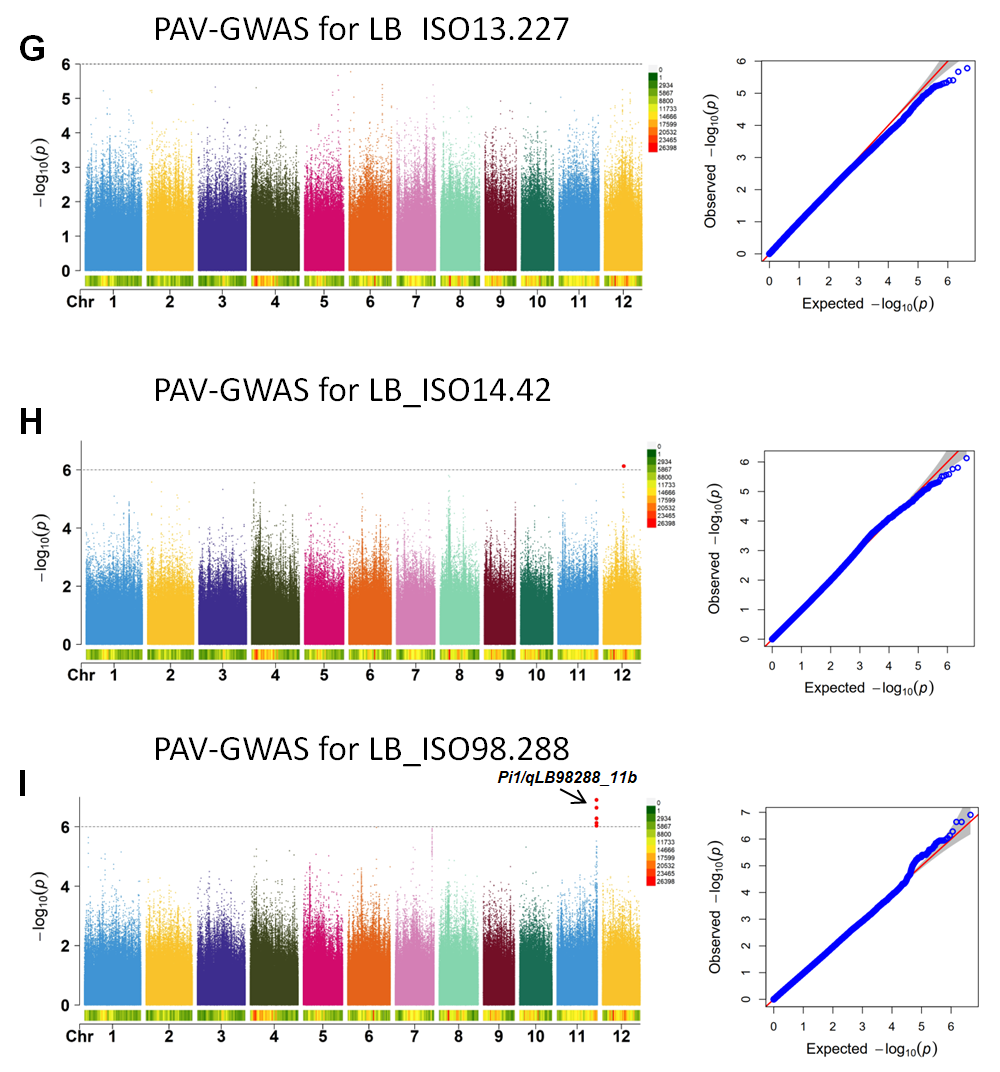


**Figure S7** Manhattan plots of the PAV-based GWAS for PBR, LBR and 7 strains resistance in 12 chromosomes and QQ plots for the PAV-GWAS


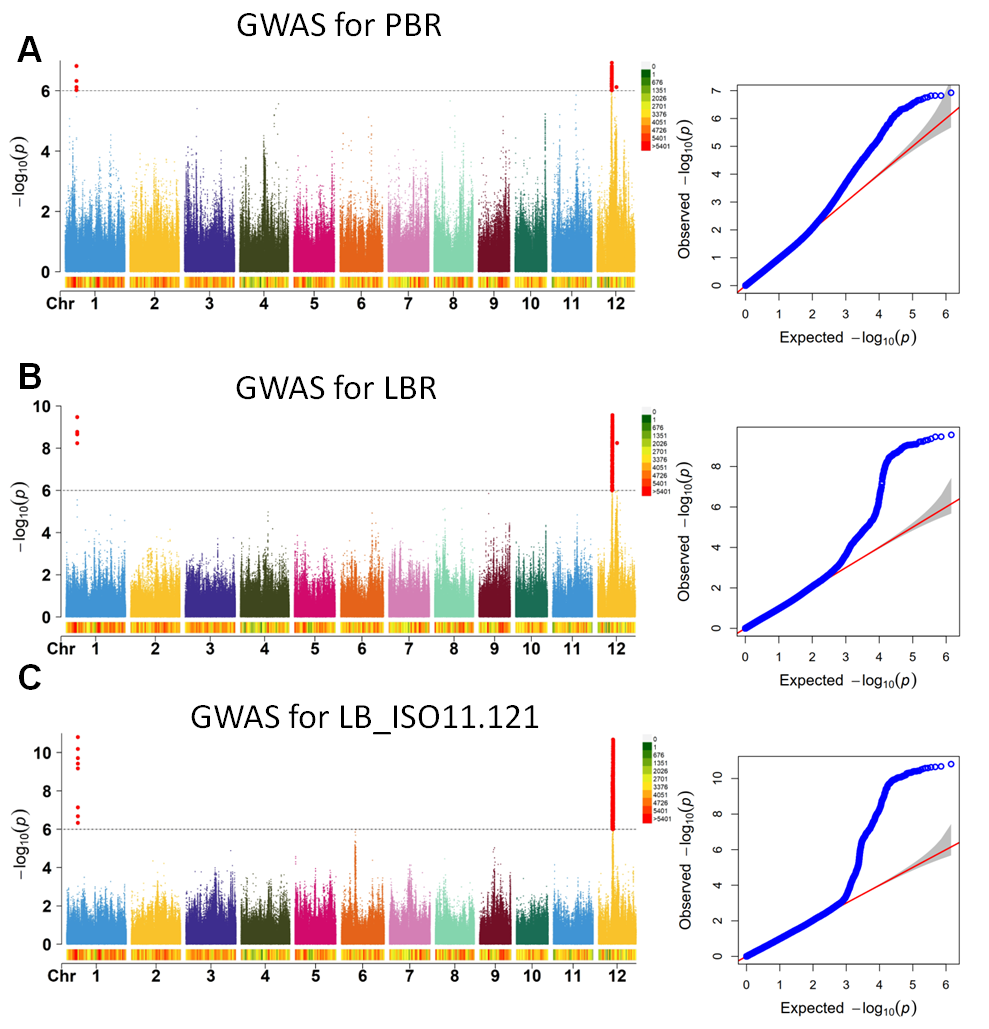

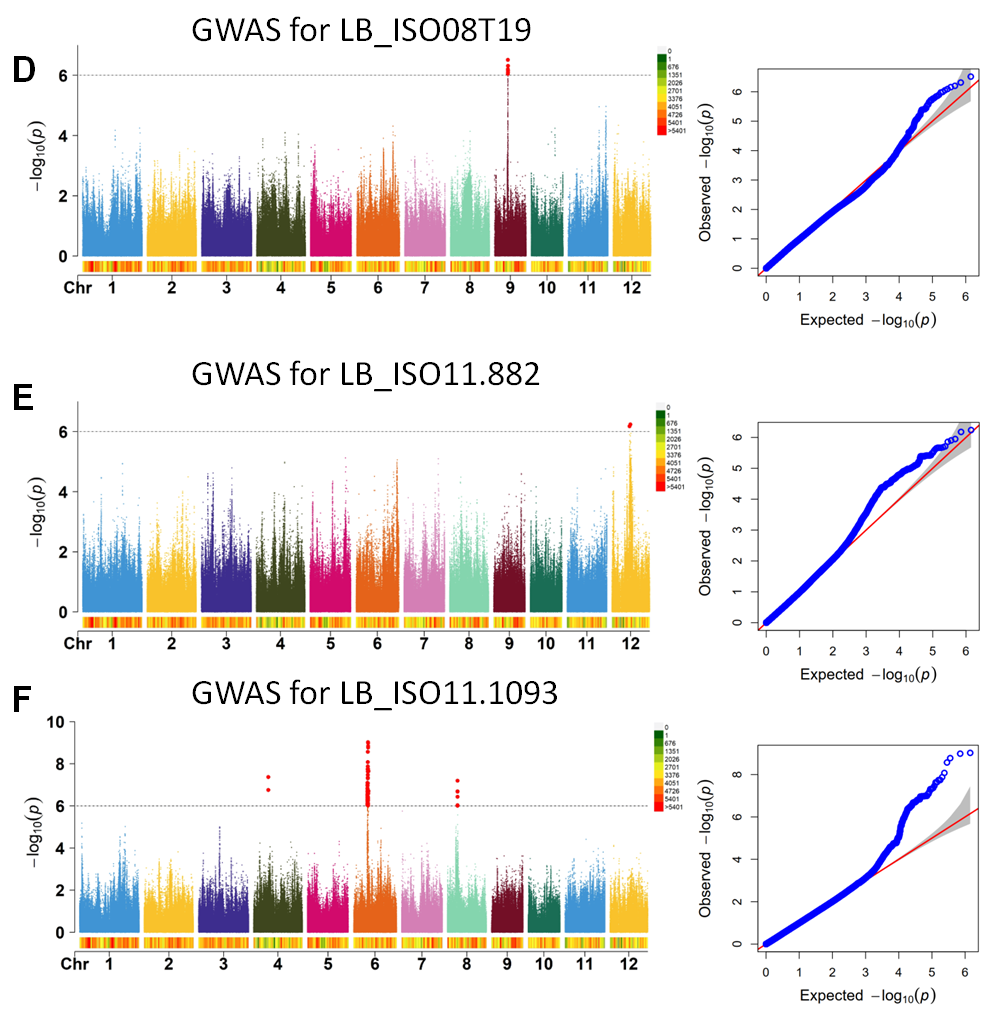

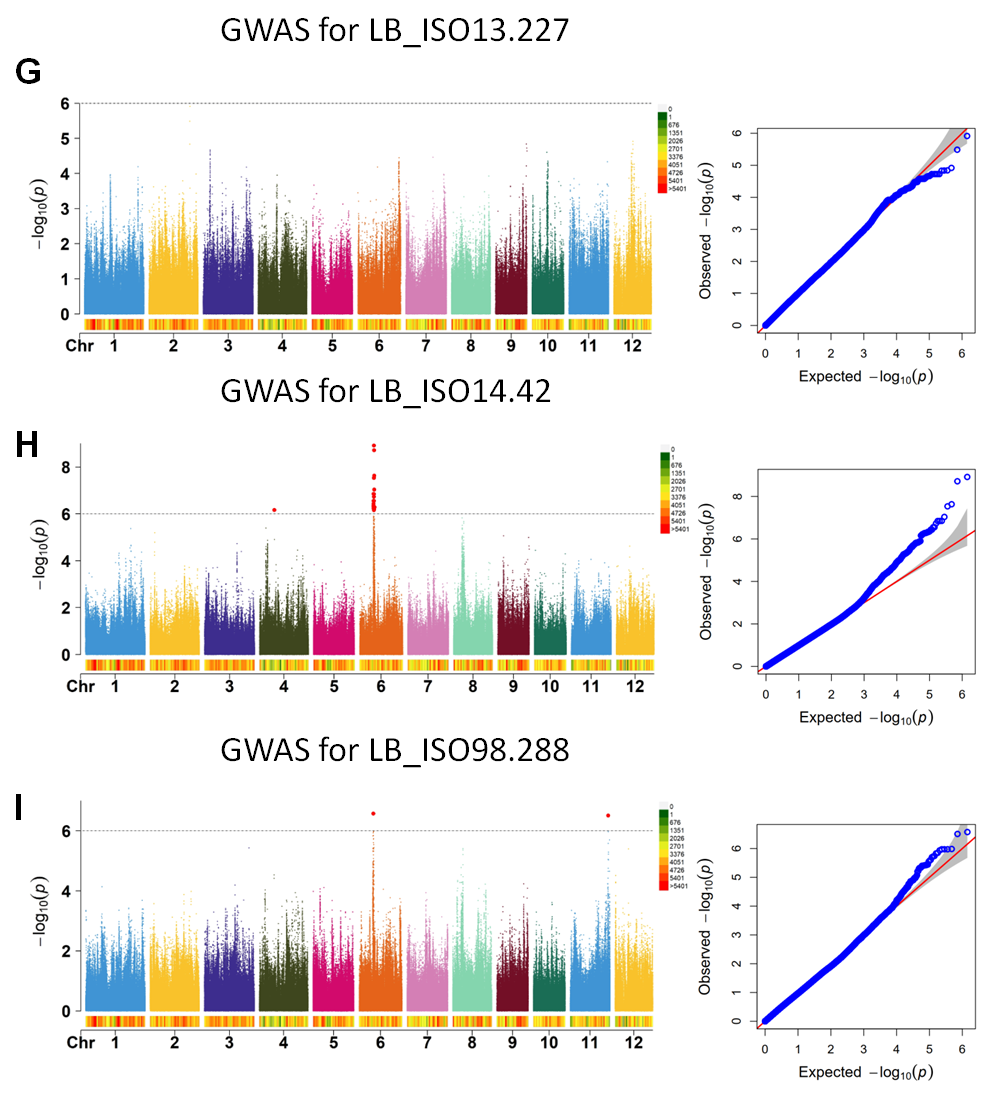


**Figure S8** Manhattan plots of GWAS with Nippnbare genome as reference genome for PBR, LBR and 7 strains resistance in 12 chromosomes and QQ plots for the GWAS


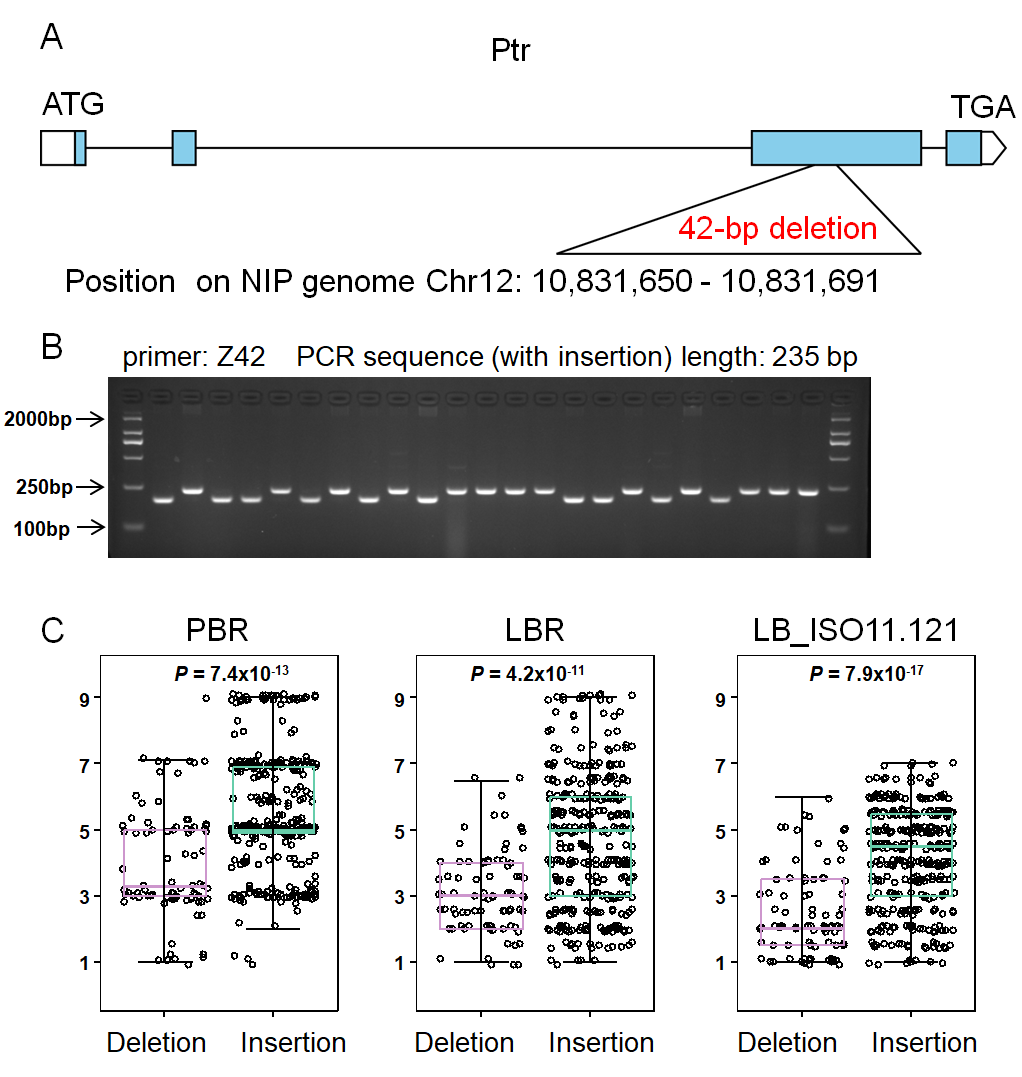


**Figure S9** 42-bp InDel at *Ptr* gene associted with blast resistance on PBR, LBR and LB_ISO11.121


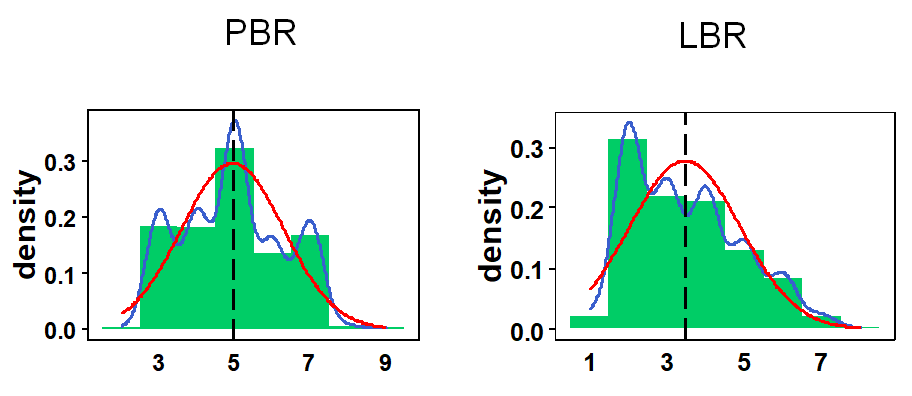


**Figure S10** Histograms of phenotype variations for panicle blast resistance (PBR) and leaf blast resistance in F_2:3_ population


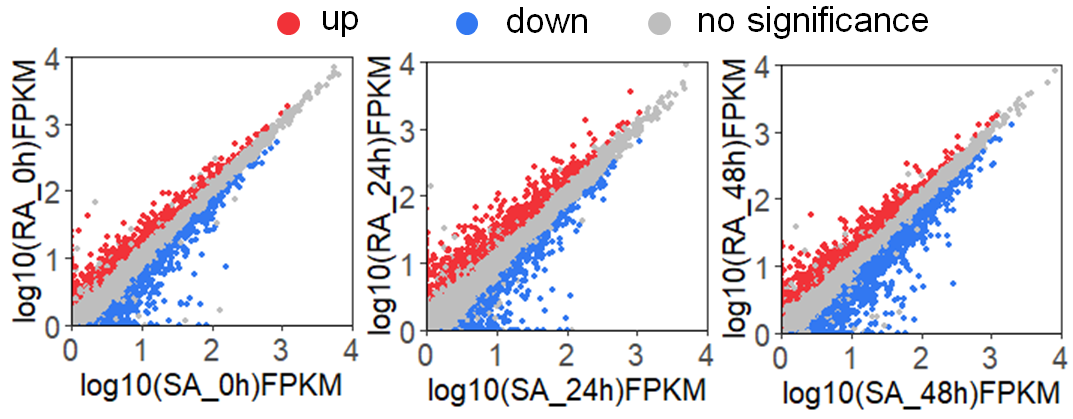


**Figure S11** Scatter plot showing the (log10 FPKM) expression of the DEGs in the three time points


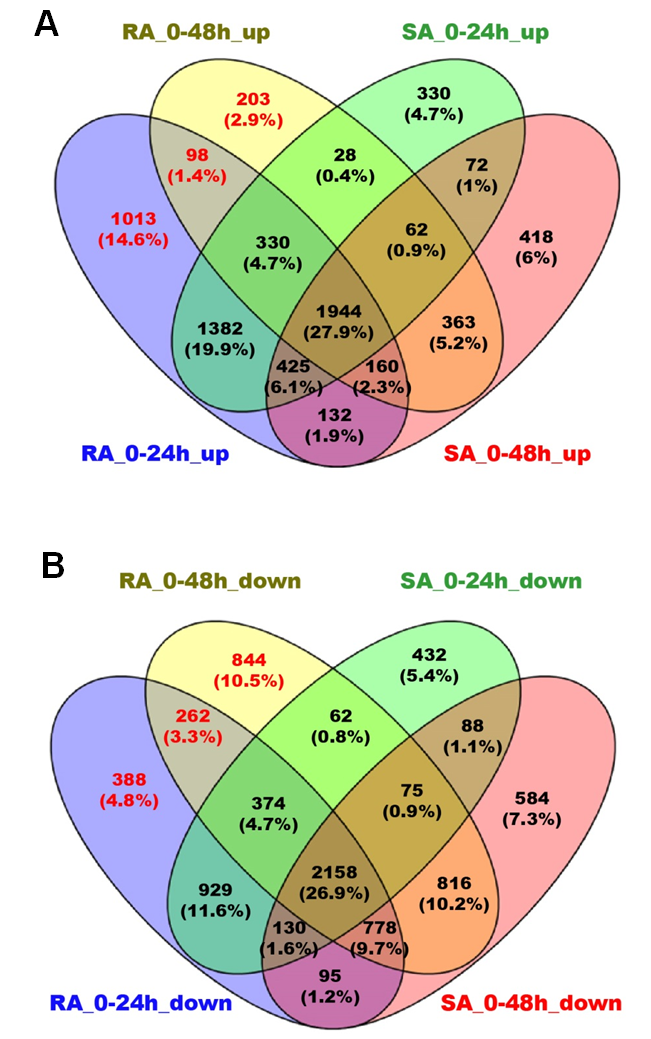


**Figure S12** Venn diagram representing the number of up- and down-regulated DEGs at 0h with those at 24h (or 48h) with rice blast fungus infection in the RA and SA


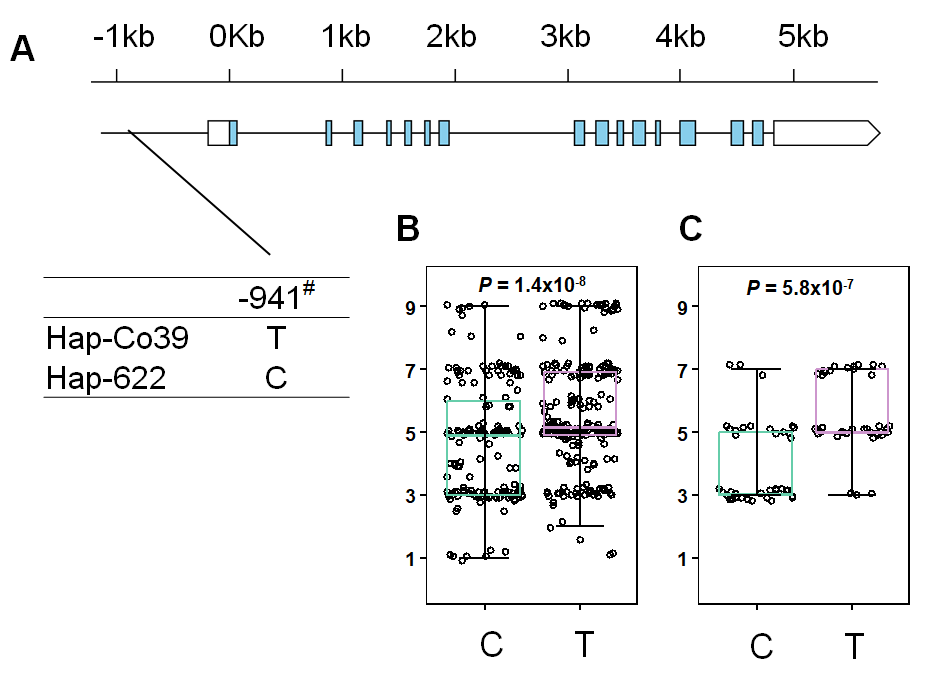


**Figure S13** Haplotype analysis of the candidate gene *LOC_Os1g14580*. a. *LOC_Os1g14580* gene structure and the separate SNP on promoter of *LOC_Os1g14580* in Co39 and 622. b. Boxplots for the PBR based on haplotypes (Hap-Co39 and Hap-622) in natural populations. c. Boxplots for the PBR based on haplotypes in Heterogeneous Inbred Family. # The SNP position is based on the initiator codon ATG of *LOC_Os1g14580*
